# Task-control adaptation in task switching: Uncovering the mechanisms behind the list-wide proportion valency effect

**DOI:** 10.3758/s13421-025-01782-1

**Published:** 2025-09-15

**Authors:** Luca Moretti, Iring Koch

**Affiliations:** Institute of Psychology, RWTH Aachen University, Jaegerstrasse 17/19, 52066 Aachen, Germany

**Keywords:** Task switching, Task conflict, Conflict adaptation, List-wide proportion of valency

## Abstract

The term conflict adaptation refers to the up- or down-regulation of control processes depending on the likelihood and recency of conflict occurrence. While most research on conflict adaptation has examined single-task settings (e.g., the Stroop paradigm), in a recent study we investigated whether similar phenomena are also present in task switching. In that study, we asked whether control over stimulus-induced task conflict is modulated depending on its frequency. We operationalized stimulus-induced task conflict with the valency effect, namely the performance difference between bivalent-congruent trials (i.e., stimuli affording two tasks but only one correct response) and univalent trials (i.e., stimuli affording one task). Manipulating the proportion of bivalent trials across blocks, we found that the valency effect was reduced in majority-bivalent blocks, suggesting that task-conflict control is upregulated under high-conflict conditions. Building on these findings, the present study presents three experiments designed to uncover the mechanisms behind task-conflict control adaptation in task switching. In particular, we aimed to assess whether task-conflict control is adapted in a transient or in a sustained fashion. In Experiment 1, we ruled out the possibility that control adaptation arises from transient increases in attentional biasing following cue onset. In Experiments 2a and 2b we found evidence that the mechanisms responsible for upregulating task-conflict control act in a task-specific fashion, suggesting that, contrary to single-task paradigms, control adaptation occurs transiently rather than sustainedly. We conclude that in task-switching contexts, conflict adaptation is achieved by modulating reactive control mechanisms triggered after stimulus onset.

**Supplementary Information:**

The online version contains supplementary material available at 10.3758/s13421-025-01782-1.

## Introduction

The term cognitive control is used to describe the set of mechanisms that are employed to achieve goal-directed behavior in the face of interference (Botvinick et al., 2001; Norman & Shallice, 1986). These mechanisms are needed under a variety of circumstances, for example when overriding prepotent responses, or when pursuing a new task in light of changes in the surrounding environment. The latter scenario has been the focus of interest of task-switching studies (for recent reviews, see Koch et al., 2018; Koch & Kiesel, 2022), where cognitive flexibility – the ability to adapt cognitive processes to environmental changes – is investigated by having participants switch between simple classification tasks, depending on the instructions of a task cue. When using this paradigm, it is consistently observed that switching bears performance costs compared to repeating the same task, providing a window into the control processes supporting cognitive flexibility (Rogers & Monsell, 1995).

Over the past two decades, there has been a growing interest in how control mechanisms are up- or down-regulated in response to contextual demands (Braver, 2012; Bugg & Crump, 2012; Egner, 2017, 2023; Gratton et al., 2018). For example, the extent to which control mechanisms supporting flexibility are engaged depends on whether the environment requires frequent switching (e.g., cooking while reading a recipe), or keeping focused on one task (e.g., reading in a crowded café). In the lab, such different environmental demands are created by manipulating the proportion of switch trials across blocks. When most trials require switching, the control processes allowing for cognitive flexibility need to be recruited more often compared to blocks where the task repeats on most trials. As a consequence, the mechanisms involved in switching are up- or down-regulated depending on the block type, as indicated by a reduction of the switch cost in majority-switch blocks (Braem et al., 2024; Dreisbach & Fröber, 2019; Fröber & Dreisbach, 2017; Geddert & Egner, 2022; Monsell & Mizon, 2006; Siqi-Liu & Egner, 2020, 2023; Strivens et al., 2024a, 2024b).

While such a list-wide proportion-switch (PS) effect has received much attention in recent years, providing valuable insights into how cognitive flexibility is regulated across different environments, little is currently known about the mechanisms regulating a related aspect of task-switching performance, namely the ability to solve task conflict induced by the stimulus. In daily life, many stimuli afford different tasks (e.g., a printer can be used for both printing and scanning), leading to conflict in task selection when such stimuli are encountered. In order to select the relevant task for our goals, this stimulus-based task conflict must be solved by control mechanisms, which we will refer to as *task-conflict control*. It is important to recognize that, although solving stimulus-based task conflict is certainly related to cognitive flexibility, the mechanisms dealing with switching have a broader scope than those solving stimulus-based task conflict (e.g., dealing with carryover activation of the previously relevant task), as outlined below. Therefore, current research focusing on the list-wide PS effect provides only indirect evidence about how we deal with stimulus-based conflict between task sets. In the present study, we aimed to fill part of this gap by investigating how task-conflict control is modulated by environmental circumstances. We will refer to this form of control adaptation as *task-control adaptation*.

## Modulations of stimulus-based task conflict in task switching

One fundamental function of cognitive control mechanisms is to solve conflict between competing cognitive representations in order to pursue our goals (Botvinick et al., 2001; Kerns et al., 2004). In task switching, cognitive conflict can occur at multiple levels and is triggered by multiple sources. In the present study, we focus on conflict at the task level, which can be elicited by two sources. First, task conflict emerges in switch trials due to the carryover activation of the previously relevant task set (Allport et al., 1994; Wylie & Allport, 2000). Such task-set inertia is beneficial in repetition trials, but leads to performance costs in switch trials, as switching requires inhibition of the previously relevant task (Gade et al., 2014; Koch et al., 2010; Mayr & Keele, 2000; Schuch & Koch, 2003). Task-set inertia is therefore a known contributor of the switch cost, together with the need to retrieve a new task-set from long-term memory (Rogers & Monsell, 1995; Rubinstein et al., 2001).

The second source of task conflict, which is the focus of the present study, is the bivalent nature of the stimuli (Koch & Allport, 2006; Waszak et al., 2003). In most task-switching paradigms, the stimuli afford both tasks. For example, colored shapes could be used in an experiment where both a color-classification and a shape-classification task are required. Presenting a bivalent stimulus (e.g., a red triangle) would therefore prime both tasks irrespective of which one is currently relevant. We refer to this type of conflict as *stimulus-based task conflict* to distinguish it from task conflict elicited by task-set inertia. If task-set inertia can be partly measured with the switch cost, in order to measure stimulus-based task conflict one needs to compare performance in bivalent trials, where the stimulus affords two tasks, with performance in univalent trials, where the stimulus only affords one task (e.g., a transparent triangle affording only the shape task). When doing so, however, it is important to distinguish stimulus-based task conflict from conflict arising at the response level. Response conflict emerges whenever the correct goal is selected (e.g., open a door) but multiple response options are available to achieve that goal (e.g., multiple keys are present; see Moretti et al., 2023). Therefore, in addition to task conflict, bivalent stimuli can also trigger conflict at the response level. This is because the responses elicited by the stimuli’s dimensions (e.g., color and shape) can either converge, in which case the trial is said to be congruent, or diverge, in which case the trial is said to be incongruent. Similar to other cognitive control tasks, performance is impaired in incongruent trials compared to congruent trials, indicating that stimulus-based response conflict takes time to be resolved (Kiesel et al., 2007; Wendt & Kiesel, 2008). Therefore, in order to gain a pure measure of stimulus-based task conflict devoid of stimulus-based response conflict, we need to compare performance in bivalent congruent trials, where the two stimuli’s dimensions elicit both task-sets but cue the same response, with performance in univalent trials, where neither task conflict nor response conflict is present. When this is done, a robust valency effect emerges, with poorer performance in bivalent congruent trials compared to univalent trials (Moretti et al., 2025; Rogers & Monsell, 1995; Steinhauser & Hübner, 2007, 2009).

In a recent study (Moretti et al., 2025, Experiment 2), we set out to investigate whether stimulus-based task conflict is modulated by contextual demands similar to what observed for the switch cost. To this aim, we manipulated the proportion of bivalent trials across blocks with half of the blocks being majority-bivalent (i.e., 75% bivalent trials) and half of the blocks being majority-univalent (i.e., 25% bivalent trials). We found the valency effect to be reduced in majority-bivalent blocks, suggesting that task-control mechanisms gained efficiency when stimulus-based task conflict occurred frequently. In the present study, we aimed at further investigating this novel list-wide proportion valency (PV) effect by elucidating how task-control mechanisms are up- or down-regulated across different blocks.

## Parallels with the Stroop literature

The results of our previous study suggest that not only cognitive flexibility (as measured with the switch cost), but also task-conflict control (as measured with the valency effect) are modulated by contextual demands in task switching. In asking how such an operation could be achieved, it may be tempting to turn to the Stroop literature where proportion effects have been widely investigated (for reviews, see Braem et al., 2019; Bugg & Crump, 2012). In the classic Stroop paradigm, participants are asked to indicate the color of a word while ignoring its meaning. Similar to task switching, it is possible to investigate stimulus-based task conflict by comparing performance in non-color words, which still elicit the irrelevant reading task but do not trigger response conflict, with univalent stimuli (e.g., a string of colored Xs; Augustinova et al., 2018, 2019; Shichel & Tzelgov, 2018). Importantly, the resulting valency effect is stronger (or only present) in majority-univalent blocks compared to majority-bivalent blocks (Entel et al., 2015; Goldfarb & Henik, 2007; Kalanthroff et al., 2018; Keha & Kalanthroff, 2023b), suggesting that task-control mechanisms can be engaged differently depending on the circumstances.

On a surface level, these findings parallel the list-wide PV effect reported in our previous study (Moretti et al., 2025). Crucially for the present study, however, the mechanisms behind such effect are likely to differ between task-switching and single-task paradigms. In the Stroop literature, there is some consensus in attributing the list-wide PV effects to a different weighting of the stimulus’ task-irrelevant feature across blocks (Kalanthroff et al., 2018; Littman et al., 2019).^1^ When most trials are bivalent, attention should be diverted away from the task-irrelevant feature in order to avoid activation from the irrelevant reading task, whereas in majority-univalent blocks attention does not need to be as selective in most of the trials. Importantly, such changes in attentional weighting are thought to be sustained (Braver, 2012), meaning that the same attentional settings are applied to all trials in a block. In task switching, however, such sustained shifts in attention to/away from the task-irrelevant feature are simply not available, given that the task-irrelevant feature constantly changes in this paradigm. For example, when switching between color and shape classification, there would be no use in inhibiting the color feature in a sustained fashion, as color will be relevant in half of the trials. Therefore, the aim of the present study is to provide a mechanistic account of the mechanisms regulating task-conflict control in task switching, as captured by the list-wide PV effect (Moretti et al., 2025). In particular, we assess the role of two candidate mechanisms: A sustained shift in task activation across blocks, and transient changes in task-set reconfiguration.

## The present study

As mentioned above, the list-wide PV effect observed in the Stroop paradigm is commonly attributed to a differential weighting of the task-irrelevant feature across blocks. Such task-control adaptation is thought to be sustained, meaning that the down- or up-regulation of task-control mechanisms is applied uniformly across trials of a block. Given the similarity between the list-wide PV effect in the Stroop paradigm and in our previous task-switching study, it may be tempting to conclude that the mechanisms behind task-control adaptation are similar across paradigms. However, it is important to recognize that sustained inhibition of the task-irrelevant feature is simply not an available strategy in task switching, as the task-irrelevant feature changes from trial to trial. For this reason, the present study seeks to provide a mechanistic account of task-control adaptation in task switching. In particular, we were interested in assessing whether task-conflict control is modulated in a transient or a sustained fashion. To this aim, we investigate the role of two possible mechanisms modulating task-conflict control, one acting transiently and one sustainedly.

The first mechanism is related to task-set preparation. When presented with the task cue, participants are thought to prepare for the upcoming trial by biasing attention toward the task-relevant feature (Logan & Gordon, 2001; Longman et al., 2013, 2014; Meiran, 2000). For example, cueing the color task would induce participants to prepare to attend the color dimension of the stimulus and ignore the irrelevant dimension. Therefore, one possible mechanism behind the list-wide PV effect is stronger attentional biasing in majority-bivalent blocks. Such a process would be transient because task preparation is accomplished on a trial-by-trial basis depending on the instructions provided by the cue. In this sense, control settings (e.g., attend color) would not stay constant throughout a block, but would rather depend on the current task at hand. In Experiment 1, we investigated whether the list-wide PV effect originates from stronger cue-based preparation in majority-bivalent blocks.

The second candidate mechanism for the list-wide PV effect is suggested by studies investigating the tradeoff between cognitive stability (the ability to shield the currently relevant task from other irrelevant tasks) and flexibility (Dreisbach & Fröber, 2019; Dreisbach & Goschke, 2004; Geddert & Egner, 2022, 2024). As mentioned above, studies manipulating the proportion of switch trials across blocks consistently report smaller switch cost in majority-switch blocks, suggesting that frequent switching promotes a more flexible mode, while frequent repetitions promote inflexibility. As flexibility is thought to trade off with task shielding (Dreisbach & Fröber, 2019; Dreisbach & Goschke, 2004; but see Geddert & Egner, 2022, 2024), poorer flexibility in majority-repetition blocks should also come with the advantage of greater task shielding (i.e., less stimulus-induced task conflict). One popular account of the list-wide PS effect holds that increased task shielding in majority-repetition blocks is achieved by activating task representations less strongly into working memory (Dreisbach & Fröber, 2019; Liu & Yeung, 2020). Following this idea, as the currently irrelevant task is less active in majority-repetition blocks, the currently relevant task should suffer less task interference (i.e., it is shielded). The same account may also explain the list-wide PV effect. From this perspective, we may hypothesize that the frequent occurrence of stimulus-based task conflict in majority-bivalent blocks would also promote task-shielding in the form of lower activation of both task sets. This mechanism would be applied in a sustained fashion, as it would act uniformly over a block of trials without the need to change control settings on a trial-by-trial basis. We investigate this possibility in Experiments 2a and 2b.

## Experiment 1

In Experiment 1 we aimed to test a transient-adaptation account of the list-wide PV effect. In particular, we asked whether task preparation is achieved to a stronger degree in majority-bivalent blocks, reducing task conflict elicited by the stimulus. We investigated task preparation by manipulating the time interval between the task cue and stimulus onset (CSI). Longer CSIs not only are found to improve overall performance, and reduce the switch cost, but also reduce the effects of stimulus-based task priming (Koch & Allport, 2006), corroborating our idea that the list-wide PV effect may arise from stronger attentional biasing in majority-bivalent blocks. Generally speaking, cue-based reconfiguration should overall be enhanced in majority-bivalent blocks as cues are not necessary to task performance when using univalent stimuli. It is therefore plausible that the list-wide PV effect observed in our previous study (Moretti et al., 2025, Experiment 2), arose from the modulation of preparatory mechanisms that take place during the relatively long CSIs chosen (700 ms).

To investigate this idea, we manipulated the CSI both within subjects (Experiment 1a) and between subjects (Experiment 1b). In both designs we expected the switch cost to be reduced when the CSI is long, signaling that participants did use the cue to prepare for the next task. If cue-based preparation is present, and if it plays a role in generating the list-wide PV effect, we predicted replicating this finding at long CSIs and, more critically, finding a reduced or even absent list-wide PV effect at short CSIs. However, when manipulating the CSI between subjects (Experiment 1b) we did not find the expected switch-cost reduction, indicating that cue-based preparation could have happened similarly across the two groups (for similar results, see Altmann, 2004; Koch, 2001; Koch & Allport, 2006). As this finding prevents us from drawing firm conclusions on the role of task-set preparation as a contributor of the list-wide PV effect, we decided to report this experiment in the Appendix. Here, we only report the results of Experiment 1a, where the CSI was manipulated within subjects.

## Experiment 1a

### Methods

#### Participants

The experiment was built and run online using Gorilla (Anwyl-Irvine et al., 2020). Sixty participants (mean age in years = 25.9, 43.5% females) were recruited through prolific and took part in the experiment in exchange for monetary compensation (20€). The sample size was determined in light of the following considerations. Our effect of interest was the three-way interaction between the within-subjects variables Valency, Block Type, and CSI. In particular, if task preparation plays a crucial role in the emergence of the list-wide PV effect, the interaction between Block Type and Valency (i.e., the list-wide PV effect) would only be present when the CSI is long, but absent at short CSIs. In other words, we predicted the list-wide PV effect to be fully attenuated at short CSI. In within-subjects designs, the effect size of a fully attenuated interaction is half of the effect that is being attenuated (Sommet et al., 2023). Based on our previous study, the interaction between Valency and Block Type was assumed to be d_z_ = 0.86. Therefore, we estimated our effect of interest to be d_z_ = 0.43. Accordingly, we aimed to include at least 45 participants in order to reach a power of 1-β = 0.80. We recruited a few more (N = 60) to compensate for possible exclusions during data trimming.

#### Tasks and stimuli

The tasks and stimuli used in this and the following experiments are those reported in previous studies from our group (Moretti et al., 2024, 2025). Strings of ten colored characters were used as bivalent stimuli. The participants’ task was either to indicate which color (red or orange) or which character (# or 0) was most present in the string. Univalent stimuli were either colored Xs, affording only the color task, or black strings of hashtags and zeros, affording only the character task. In both bivalent and univalent stimuli, the ratio between elements of each feature was always kept to 6:4. For example, in a bivalent stimulus there may be six red characters and four orange characters, and six hashtags and four zeros. This choice was made to equate saliency of the task-relevant and task-irrelevant features, as stimulus-based task conflict was found to be larger when the task-irrelevant feature is more salient than the task-relevant feature (Moretti et al., 2024).

Each trial started with the presentation of a task cue at the center of the screen. The drawing of a sun and the drawing of a cloud were used to this purpose, with their relation to the tasks counterbalanced across participants. The task cue stayed on-screen for either 200 or 1,200 ms before being replaced by the stimulus. Whether the CSI was short or long varied randomly from trial to trial. After stimulus onset, participants had a maximum of 2,000 ms to provide a response by pressing the “A” or “L” keys of the keyboard. After this time elapsed, or when a response was provided, the screen turned blank for 200 ms (if the CSI was 1,200 ms in the following trial) or 1,200 ms (if the CSI was 200 ms in the following trial). Therefore, the interval between stimuli was kept constant (i.e., 1,400 ms) to control for decay of the previously relevant task (for a recent review, see Koch & Kiesel, 2022).

#### Experimental procedure

The experiment was divided in two sessions separated by 1 to 7 days. Each session started with two practice blocks of 16 trials during which participants were familiarized with the tasks. After these blocks, a mixed block of 64 trials introduced the task-switching procedure. During the whole practice phase, performance feedback was provided after an erroneous response or if the response time (RT) exceeded 2,000 ms. Following practice, eight blocks of 128 trials were administered in each session. In one session, the blocks were either all majority-bivalent (i.e., 75% of the trials were bivalent) or all majority-univalent (i.e., 25% of the trials were bivalent). In the other session the other block type was presented. The order of majority-bivalent and majority-univalent sessions was counterbalanced across participants.

The sequence of tasks and stimuli in each block was determined pseudo-randomly with the constraints that there would be an equal number of trials for each combination of task (color or character), task sequence (switch or repeat), CSI (200 ms or 1,200 ms) and, among bivalent trials, congruency (congruent or incongruent). The proportion of univalent/bivalent trials was determined depending on the identity of the block as described above. Similarly, the proportion of trials following a univalent/bivalent trial followed the same rules. The resulting trial tree is depicted in Fig. S2 of the Online Supplementary Materials.

#### Statistical design

Separate 2 × 2 × 2 × 2 within-subjects ANOVAs were conducted on RTs and arcsine transformed error rates (Laurencelle & Cousineau, 2023). In both ANOVAs the independent variables were Valency (Bivalent, Univalent), Block Type (Majority-bivalent, Majority-univalent), CSI (Long, Short), and Task Sequence (Repetition, Switch). Our effect of main interest was the three-way interaction between Valency, Block Type, and CSI. If task preparation is crucial for reducing stimulus-based task conflict in majority-bivalent blocks, this interaction should indicate that the list-wide PV effect is only present in the long-CSI group. We had no specific predictions concerning the effects of task switching on our interaction of interest (i.e., we had no predictions on the four-way interaction). Nonetheless, Task Sequence was included in the design for two reasons. First, it seems plausible that switching may modulate the list-wide PV effect. Indeed, in our previous study we did observe this effect to be larger in switch trials compared to repetition trials (Moretti et al., 2025). Second, we wanted to check that participants use the CSI for preparing. If this is the case, we should observe better overall performance and smaller switch costs in the long-CSI condition.

For the sake of brevity, in each experiment we only report significant interactions in the main text. Complete ANOVA results can be found in the Online Supplementary Materials. Significant interactions were further explored by creating separate datasets for each level of a factor and running separate ANOVAs on those datasets. Although not pre-registered, as some of our predictions involve null effects (e.g., the list-wide PV effect should be absent in the short-CSI condition), we also performed Bayesian ANOVAs when decomposing interactions.^2^ Bayes factors were calculated in two stages using the BayesFactor package for R (version 0.9.12). First, for each set of independent variables (e.g., A and B), we created different models for all possible combinations of effects (i.e., all combinations of main effects and interactions). This implied that, contrary to the default implemented in the Bayes Factor package, some models also included interaction terms without necessarilly including lower-order effects (e.g., models including the A × B interaction did not necessarily include both main effects of A and B). Bayes factors were then calculated as the ratio of a model’s likelihood to that of the intercept-only model. If the proportional error estimate on the Bayes factor was above 5%, we ran the ANOVA again increasing the number of Monte Carlo samples (the default in the BayesFactor pacakge is 10,000 samples). Next, inference on a specific effect of interest was conducted by comparing the Bayes factor of the best-fitting model (i.e., the model with the highest Bayes factor relative to the intercept-only model) to that of a similar model differing only in the inclusion or exclusion of the effect of interest. For instance, if the best-fitting model included the main effect of A and the A × B interaction, evidence for the (null) main effect of B was assessed by calculating the ratio of this model’s Bayes factor to that of the full model (Rouder et al., 2017).

### Results

#### Data trimming

Before analyzing the data, we excluded participants whose error rate was higher than 33% (one participant), or providing suspiciously fast responses (RT < 200 ms) in more than 10% of the trials (no participants). The remaining fast responses were discarded from analysis, along with the subsequent trial.

On the trial level, we excluded all incongruent trials in order to measure the valency effect by comparing congruent and univalent trials. This allowed us to isolate task conflict from response conflict (Goldfarb & Henik, 2007). In addition, as the valency effect is reduced following bivalent trials (Moretti et al., 2025), we excluded all trials following a bivalent trial. This is because, in majority-bivalent blocks, trials are more likely to follow a bivalent trial, thus inflating the list-wide PV effect. In other words, we made sure that the observed list-wide PV effect is not a result of local control adjustments (i.e., the effects of N-1 valency on the current trial), but rather global adjustments (i.e., the effect of the list-wide proportion-valency manipulation). Note that excluding both incongruent and N-1 bivalent trials led to an exclusion of 59.4% of all trials by design. Finally, we excluded the first trial of each block, timeout trials, trials following a timeout, and trials following an error. Errors were excluded from the RT analysis, but retained for the error rates analysis. At the end of data trimming we retained 31.4% of the trials for the included participants. The mean number of survived trials in each cell of the design per participant was 40.2 (SD = 43.0).

#### Response time (RT) analyses

**Fig. 1:**
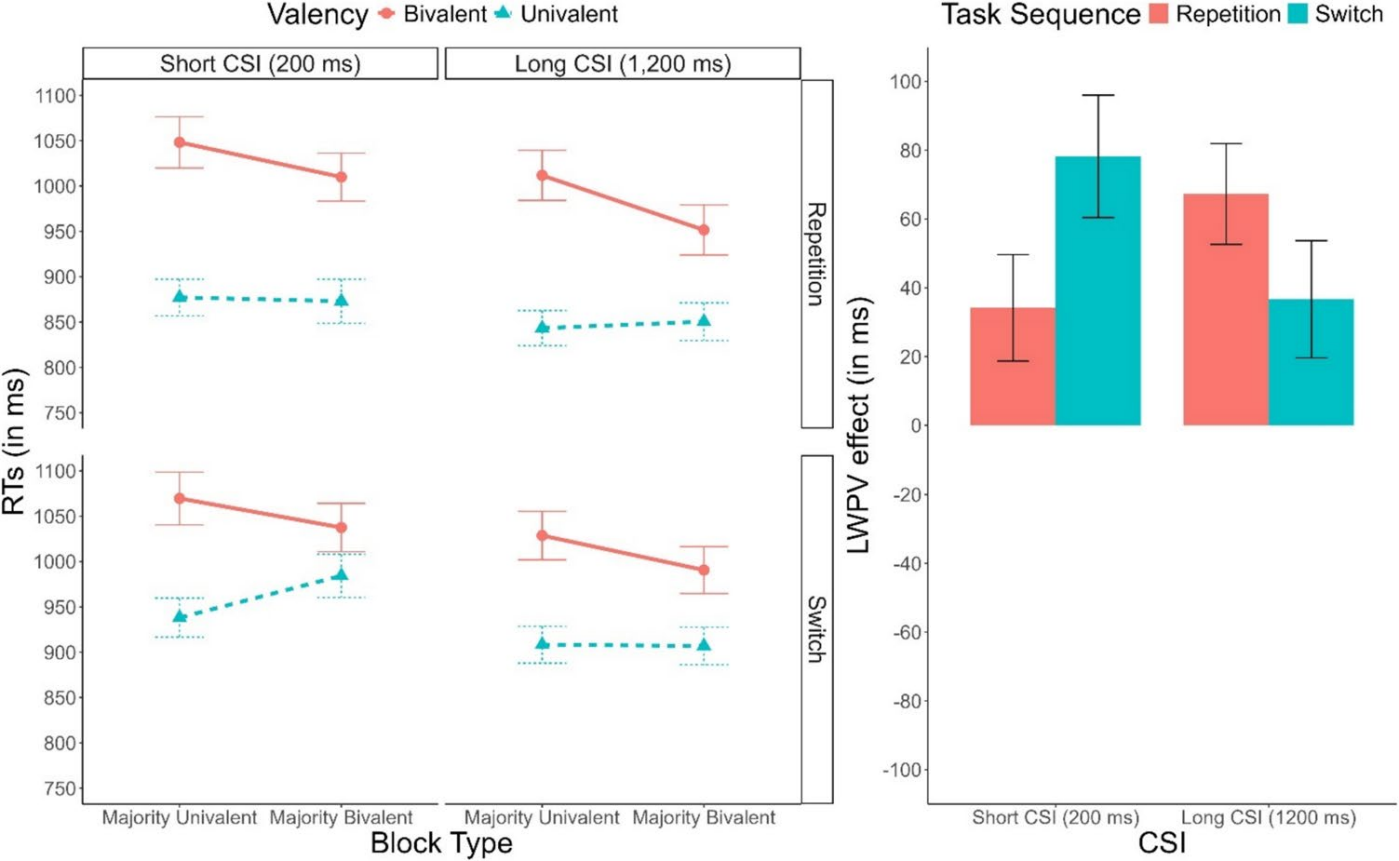
**Left panel:** Mean response time (RT) as a function of CSI (Short, Long), Task Sequence (Repetition, Switch), Valency (Bivalent, Univalent) and Block Type (Majority-Univalent, Majority-Bivalent). **Right panel:** Mean list-wide proportion of valency effect as a function of Task Sequence and CSI. Error bars represent the standard error of the mean

Descriptive statistics of RTs are plotted in Fig. 1. The ANOVA revealed main effects of Task Sequence, *F*(1, 58) = 72.85, *p* <.001, ${\upeta }_{p}^{2}$ =.56, ${\upeta }_{G}^{2}$ =.02, and Valency, *F*(1, 58) = 146.44, *p* <.001, ${\upeta }_{p}^{2}$ =.72, ${\upeta }_{G}^{2}$ =.10, indicating a robust switch cost and valency effect, respectively. These two factors interacted significantly, *F*(1, 58) = 54.74, *p* <.001, ${\upeta }_{p}^{2}$ =.49, ${\upeta }_{G}^{2}$ <.01, indicating a larger valency effect in repetition trials (144 ms), compared to switch trials (98 ms). Importantly, the main effect of CSI was also significant, *F*(1, 58) = 36.31, *p* <.001, ${\upeta }_{p}^{2}$ =.39, ${\upeta }_{G}^{2}$ =.01, indicating faster performance in the long CSI condition compared to the short CSI condition. Valency interacted with Block Type, *F*(1, 58) = 35.40, *p* <.001, ${\upeta }_{p}^{2}$ =.38, ${\upeta }_{G}^{2}$ =.01, revealing the presence of the list-wide PV effect, with a smaller valency effect in majority-bivalent blocks (93 ms) compared to majority-univalent blocks (150 ms).

Finally, we observed a four-way interaction involving all factors, *F*(1, 58) = 6.34, *p* =.015, ${\upeta }_{p}^{2}$ =.10, ${\upeta }_{G}^{2}$ <.01. To decompose this effect, we run separate three-way ANOVAs for each level of Task Sequence. In repetition trials, the significant interaction between Valency and Block Type, *F*(1, 58) = 21.77, *p* <.001, ${\upeta }_{p}^{2}$=.27, ${\upeta }_{G}^{2}$ <.01, BF_10_ = 6.19, indicated the presence of a list-wide PV effect. However, this effect was not significantly modulated by CSI, as indicated by anecdotical evidence in favor of the null hypothesis, *F*(1, 58) = 2.51, *p* =.118, ${\upeta }_{p}^{2}$ =.04, ${\upeta }_{G}^{2}$ <.01, BF_10_ = 0.30. In switch trials, we also found a list-wide PV effect, *F*(1, 58) = 20.85, *p* <.001, ${\upeta }_{p}^{2}$=.26, ${\upeta }_{G}^{2}$ <.01, BF_10_ = 11.2, but again, this effect was not significantly modulated by CSI, *F*(1, 58) = 2.98, *p* =.090, ${\upeta }_{p}^{2}$=.05, ${\upeta }_{G}^{2}$ <.01, BF_10_ = 0.33. The observed four-way interaction was therefore driven by the opposite effects that CSI had in switch and repetition trials, although in neither case did this effect reach significance. While in repetition trials the list-wide PV effect was numerically smaller in the short CSI condition (34 ms) compared to the long CSI condition (67 ms), in switch trials the list-wide PV effect was numerically larger in the short CSI condition (78 ms) compared to the long CSI condition (37 ms).

#### Error rate analyses

**Fig. 2:**
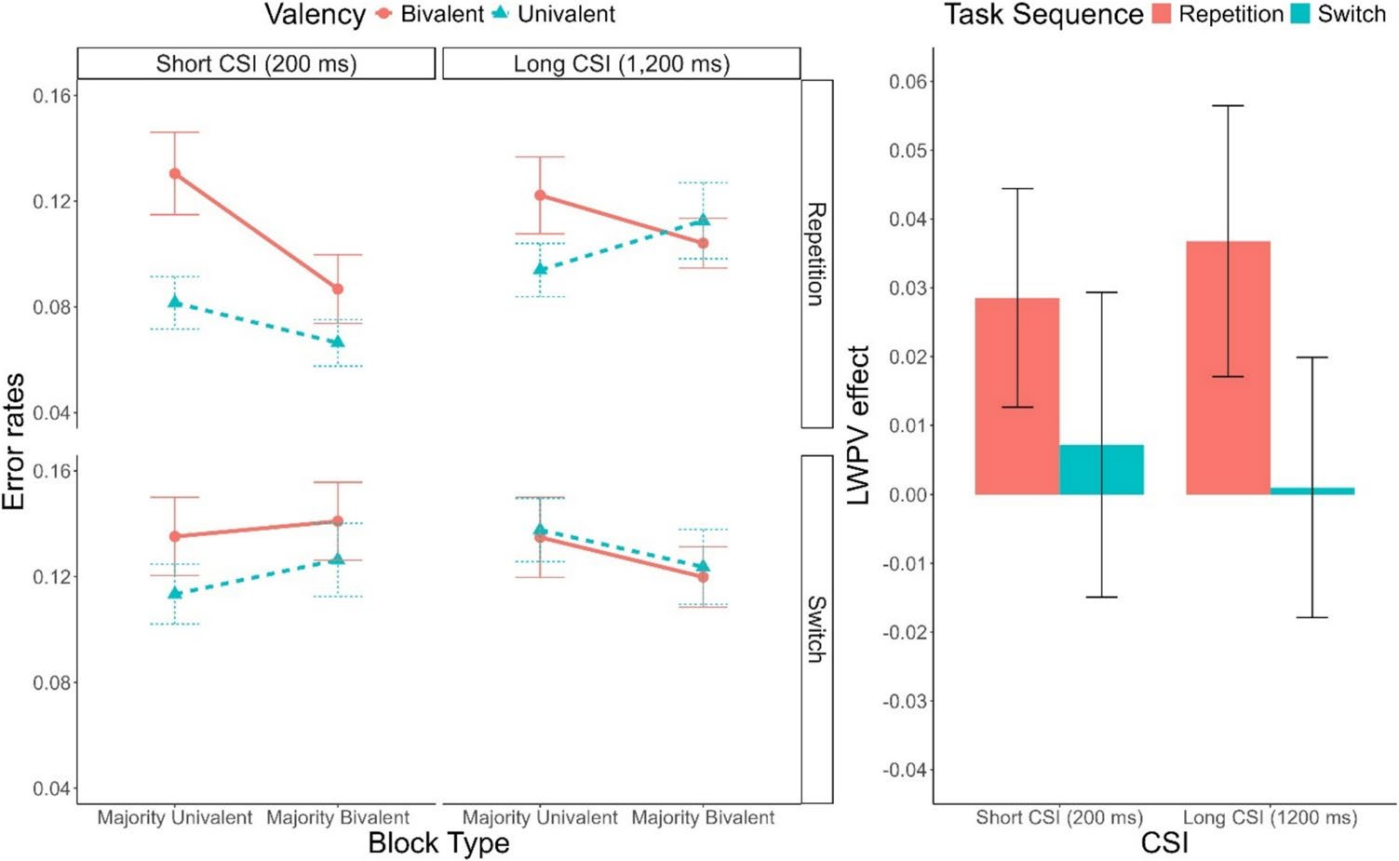
**Left panel:** Mean error rate as a function of CSI (Short, Long), Task Sequence Repetition, Switch), Valency (Bivalent, Univalent) and Block Type (Majority-Univalent, Majority-Bivalent). **Right panel:** Mean list-wide proportion of valency effect as a function of Task Sequence and CSI. Error bars represent the standard error of the mean

Descriptive statistics of the error rates are plotted in Fig. 2. In the error rates analyses, we found significant main effects of Block Type, *F*(1, 58) = 4.42, *p* =.040, ${\upeta }_{p}^{2}$ =.07, ${\upeta }_{G}^{2}$ <.01, indicating higher error commission in majority-univalent blocks compared to majority bivalent blocks. Furthermore, the main effect of Task Sequence, *F*(1, 58) = 32.73, *p* <.001, ${\upeta }_{p}^{2}$ =.36, ${\upeta }_{G}^{2}$ =.04, indicated the emergence of switch cost. Importantly, Task Sequence interacted with CSI, *F*(1, 58) = 5.43, *p* =.023, ${\upeta }_{p}^{2}$ =.09, ${\upeta }_{G}^{2}$ <.01, revealing a preparation effect with a smaller switch cost in the long CSI trials (2.1%) compared to the short CSI trials (3.8%). This interaction was further characterized by Block Type, *F*(1, 58) = 12.27, *p* <.001, ${\upeta }_{p}^{2}$ =.17, ${\upeta }_{G}^{2}$ =.01. While the preparation effect was indeed significant in majority-bivalent blocks, *F*(1, 58) = 12.78, *p* <.001, ${\upeta }_{p}^{2}$ =.18, ${\upeta }_{G}^{2}$ =.05, BF_10_ = 11.56, no such effect emerged in majority-univalent blocks, *F*(1, 58) < 1, BF_10_ = 0.18.

### Discussion

In Experiment 1 we tested the hypothesis that task-control adaptation is achieved by strengthening cue-based preparation when task conflict is frequent (i.e., in majority-bivalent blocks). To this aim, we manipulated the CSI both within participants (Experiment 1a) and between participants (Experiment 1b, reported in the Appendix), so that cue-based preparation could only take place when the CSI was long. If task-control adaptation is driven by differences in preparation across block types, we should expect to observe its effects only when preparation can take place (i.e., at long CSI).

Since in Experiment 1b we did not find preparation effects, thus preventing us to establish a clear link between cue-based preparation and the list-wide PV effect, we decided to report its results in the Appendix. Here we focus on the results of Experiment 1a, where we found evidence that participants did exploit the task cues. This was indicated in the RTs by an overall faster performance at long CSIs, and in the error rates by a reduction of the switch cost. In the Appendix we discuss in more detail preparation effects in our study, and the established differences between within-subjects and between-subjects designs, with within-subjects designs being generally more effective in eliciting preparation (Altmann, 2004; Koch, 2001). For the moment, however, we just wish to point out that finding cue-based preparation in Experiment 1a allowed us to assess whether this played a role in the emergence of the list-wide PV effect. Overall, our results suggest that this would not be the case. In Experiment 1a the three-way interaction between Block Type, Valency, and CSI did not reach significance either in RTs or in error rates (*F*s < 1). Although in the RT analysis we observed a four-way interaction involving all factors, the CSI manipulation did not significantly modulate the list-wide PV effect either in repetition or in switch trials. Collectively, these results suggest that task-control adaptation is not achieved by modulating cue-based preparation across blocks. In Experiment 2, we turn to test a sustained account of task-control adaptation.

## Experiment 2

In Experiment 1 we aimed to test a transient control-adaptation account of the list-wide PV effect, failing to provide support for the idea that task-set preparation plays a role in its emergence. In Experiment 2 we turn to testing a sustained control-adaptation account. If task control is adapted in a sustained fashion, the list-wide PV effect should be noticeable throughout a block of trials, irrespective of trial-by-trial variations (e.g., which task or stimulus is currently employed). For example, it would be possible that task sets are kept in a less active state throughout majority-bivalent blocks (cf. Dreisbach & Fröber, 2019). This would facilitate shielding against the currently irrelevant task, thus reducing task conflict. If this mechanism acts in a sustained fashion, it would not matter which task is currently irrelevant: Its activation level would be low throughout majority-bivalent blocks to avoid interference with the currently relevant task.

In Experiment 2, we tested this prediction by associating each of two tasks with a different likelihood of encountering a bivalent stimulus. Majority-bivalent and majority-univalent blocks were created by associating one task with 75%/25% occurrence of bivalent stimuli. We refer to this task as the inducer task, as it induces the block to be majority bivalent or majority univalent (cf. Braem et al., 2019). In the other task instead, the probability of encountering a bivalent stimulus was kept at 50% across blocks. We refer to this task as the diagnostic task, as it allows us to establish whether varying the proportion of bivalent trials across blocks also affects task-conflict control when performing a task that is not differently associated with bivalent and univalent stimuli. In other words, this set-up allows to test whether the list-wide PV effect originates from the global probability of task-conflict occurrence in a block, or rather from local mechanisms that are put in place depending on the task at hand. If the former is true, the list-wide PV effect should emerge in both the inducer and the diagnostic tasks. If instead the occurrence of the list-wide PV effect depends on mechanisms regulating control locally, this effect should only be observed for the inducer task.

## Experiment 2a

### Methods

#### Participants

Experiment 2a was pre-registered at: https://aspredicted.org/ZNW_J49. Similar to Experiment 1a, our main theoretical focus lay in the modulation of the list-wide PV effect by a within-subject factor. In Experiment 1a, a transient control-adaptation account would have predicted the list-wide PV effect to be present at long CSIs, but absent at short CSIs. Here, a sustained control-adaptation account would predict the list-wide PV effect to be present in both the diagnostic and the inducer task. In order to be able to discredit this idea, we therefore need to recruit enough participants to observe a three-way interaction between Block Type, Valency, and Task Type. As explained in Experiment 1a, as this interaction would be half of the size of the list-wide PV effect, our estimate of interest was again d_z_ = 0.43. To detect this effect with 80% power, we therefore needed to recruit 45 participants. We decided to recruit some more to compensate for possible data-loss during data trimming. Sixty students from RWTH Aachen University (mean age = 21.0 years, 90% females) took part in the experiment in exchange for partial course credits. The final sample size following data trimming was *N* = 54 (see below).

#### Experimental procedure

The methods were identical to Experiment 1a with two exceptions. First, the CSI and the response-cue interval were kept constant throughout the experiment. The CSI was kept at 1,200 ms (as in the long CSI condition of Experiment 1a), whereas the response-cue interval was kept at 200 ms. Second, the proportion of bivalent trials changed for only one of the two tasks across blocks. We refer to this task as the *inducer task*, because it induces the block to be majority bivalent or majority univalent. When the inducer task was relevant, the probability of encountering a bivalent stimulus was 75% in majority-bivalent blocks, and 25% in majority-univalent blocks. In the other task instead, the probability of encountering a bivalent stimulus was always 50%, irrespective of the block type. We refer to this task as the *diagnostic task*, as it allows us to test whether the list-wide PV effect is present irrespective of the specific task at hand. With this set-up, the global proportion of bivalent trials was 62.5% in majority-bivalent blocks and 37.5% in majority-univalent blocks.

The identity of the inducer and diagnostic tasks stayed the same across sessions and was counterbalanced between participants. The sequence of tasks and stimuli in each block was determined pseudo randomly with the constraint that there would be an equal number of trials for each combination of task (color or character), task sequence (switch or repeat), and, among bivalent trials, congruency (congruent or incongruent). The proportion of univalent/bivalent trials was determined depending on the identity of the block and of the task, as described above. Similarly, the proportion of trials following a univalent/bivalent trial followed the same rules. The resulting trial tree is depicted in Fig. S3 of the Online Supplementary Materials.

#### Statistical design and data trimming

Data trimming was very similar to Experiment 1a, with the only exception that participants’ data were excluded if they had less than ten trials in each cell of the experimental design (for a better overview of the cell sizes in each condition, see Fig. S3 in the Online Supplementary Materials). Data from four participants were excluded based on this criterion. In addition, two participants were excluded due to having an error rate above 33%, and none due to providing suspiciously fast responses (RT < 200 ms) in over 10% of the trials. On the trial level, data trimming was identical to Experiment 1a. The number of analyzable trials by design was 44.5%. At the end of data trimming we retained 31.5% of the trials. The mean number of survived trials in each cell of the design per participant was 40.3 (SD = 29.9).

Following data trimming, data were submitted to separate 2 × 2 × 2 × 2 within-subjects ANOVAs, conducted on RTs and arcsine transformed error rates (Laurencelle & Cousineau, 2023). In both ANOVAs the independent variables were Valency (Bivalent, Univalent), Block Type (Majority-bivalent, Majority-univalent), Task Type (Inducer, Diagnostic), and Task Sequence (Repetition, Switch). Our effect of main interest was the three-way interaction between Valency, Block Type, and Task Type, allowing us to assess whether the list-wide PV effect was limited to the inducer task.

### Results

#### RT analyses

**Fig. 3:**
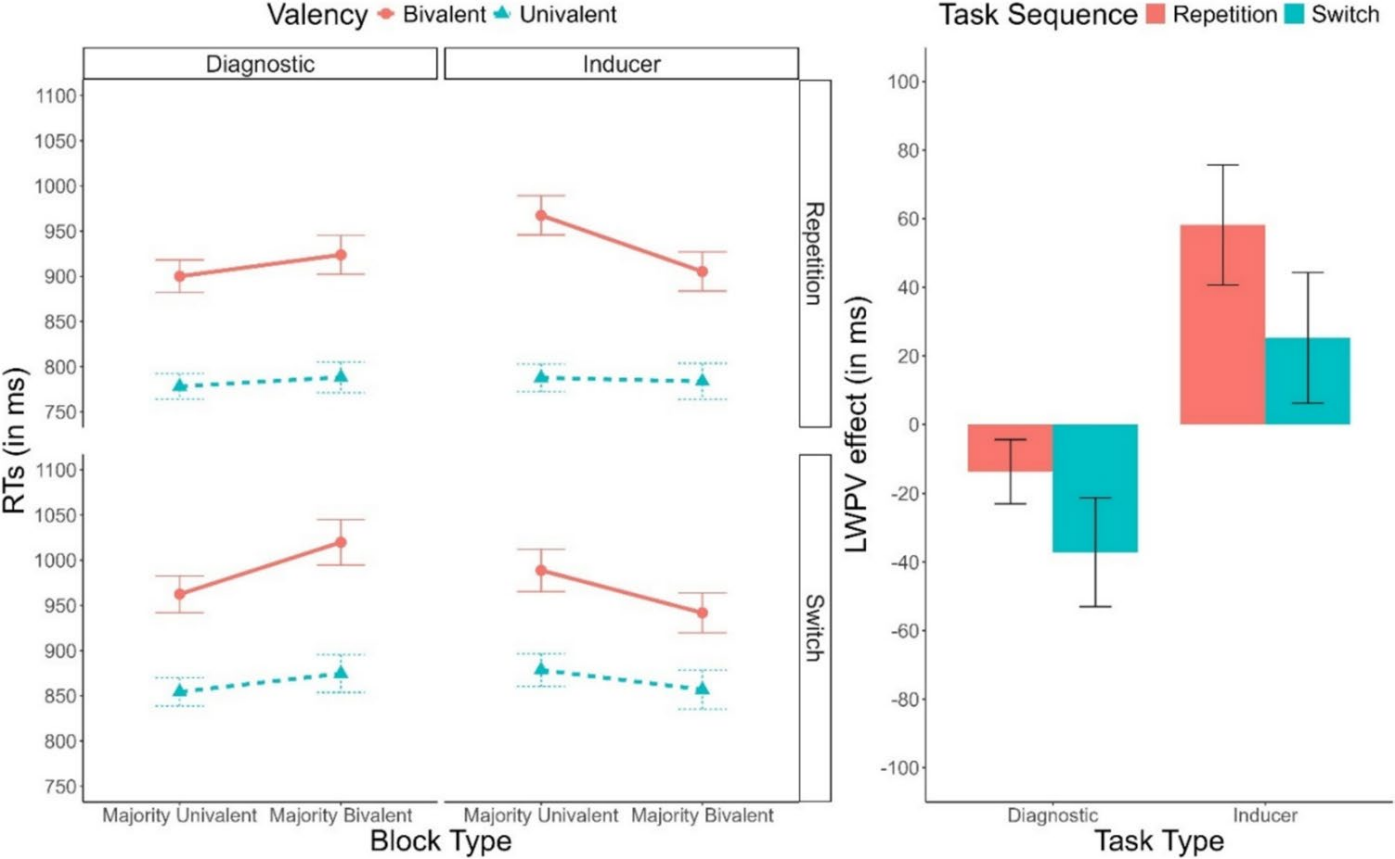
**Left panel:** Mean response times (RTs) as a function of Task Type (Inducer, Diagnostic), Task Sequence (Repetition, Switch), Valency (Bivalent, Univalent), and Block Type (Majority-Univalent, Majority-Bivalent). **Right panel:** List-wide proportion of valency (LWPV) effect as a function of Task Sequence and Task Type. Error bars represent the standard error of the mean

Descriptive statistics of the RTs are depicted in Fig. 3. As in Experiment 1a, only significant effects are reported for the sake of conciseness. Tables reporting all results can be found in the Online Supplementary Materials. We found main effects of Task Sequence, *F*(1, 53) = 129.90, *p* <.001, ${\upeta }_{p}^{2}$ =.71, ${\upeta }_{G}^{2}$ =.05, indicating a significant switch cost, and Valency, *F*(1, 53) = 220.50, *p* <.001, ${\upeta }_{p}^{2}$ =.81, ${\upeta }_{G}^{2}$ =.15, indicating a significant valency effect. These two factors interacted, *F*(1, 53) = 12.02, *p* =.001, ${\upeta }_{p}^{2}$ =.18, ${\upeta }_{G}^{2}$ <.01, with a larger valency effect in repetition trials (140 ms) than in switch trials (112 ms). This interaction was, however, further modulated by Task Type, *F*(1, 53) = 9.07, *p* =.004, ${\upeta }_{p}^{2}$ =.15, ${\upeta }_{G}^{2}$ <.01, reaching significance only in the inducer task, *F*(1, 53) = 17.87, *p* <.001, ${\upeta }_{p}^{2}$ =.25, ${\upeta }_{G}^{2}$ =.01, BF_10_ = 8.00, not in the diagnostic task, *F*(1, 53) < 1, BF_10_ = 0.14. Furthermore, Block Type significantly interacted with Task Type, *F*(1, 53) = 48.84, *p* <.001, ${\upeta }_{p}^{2}$ =.48, ${\upeta }_{G}^{2}$ =.01, indicating that, while for the inducer task performance was faster in majority-bivalent blocks (34 ms), in the diagnostic task the opposite pattern was observed (− 28 ms). Importantly, this interaction was further modulated by Valency, *F*(1, 53) = 15.52, *p* <.001, ${\upeta }_{p}^{2}$ =.23, ${\upeta }_{G}^{2}$ <.01. In the inducer task the two-way interaction between Valency and Block Type indicated a significantly reduced valency effect in majority-bivalent blocks (i.e., a list-wide PV effect), *F*(1, 53) = 9.09, *p* =.004, ${\upeta }_{p}^{2}$ =.15, ${\upeta }_{G}^{2}$ =.01. However, the corresponding Bayes factor (BF_10_ = 1.58) provided only anecdotal support for this effect. In the diagnostic task, the list-wide PV effect was reversed, as the valency effect was *larger* in majority-univalent blocks *F*(1, 53) = 6.88, *p* =.011, ${\upeta }_{p}^{2}$ =.11, ${\upeta }_{G}^{2}$ <.01. However, again, the Bayesian analyses (BF_10_ = 0.34) did not lend support to the presence of this effect.

#### Error rate analyses

**Fig. 4:**
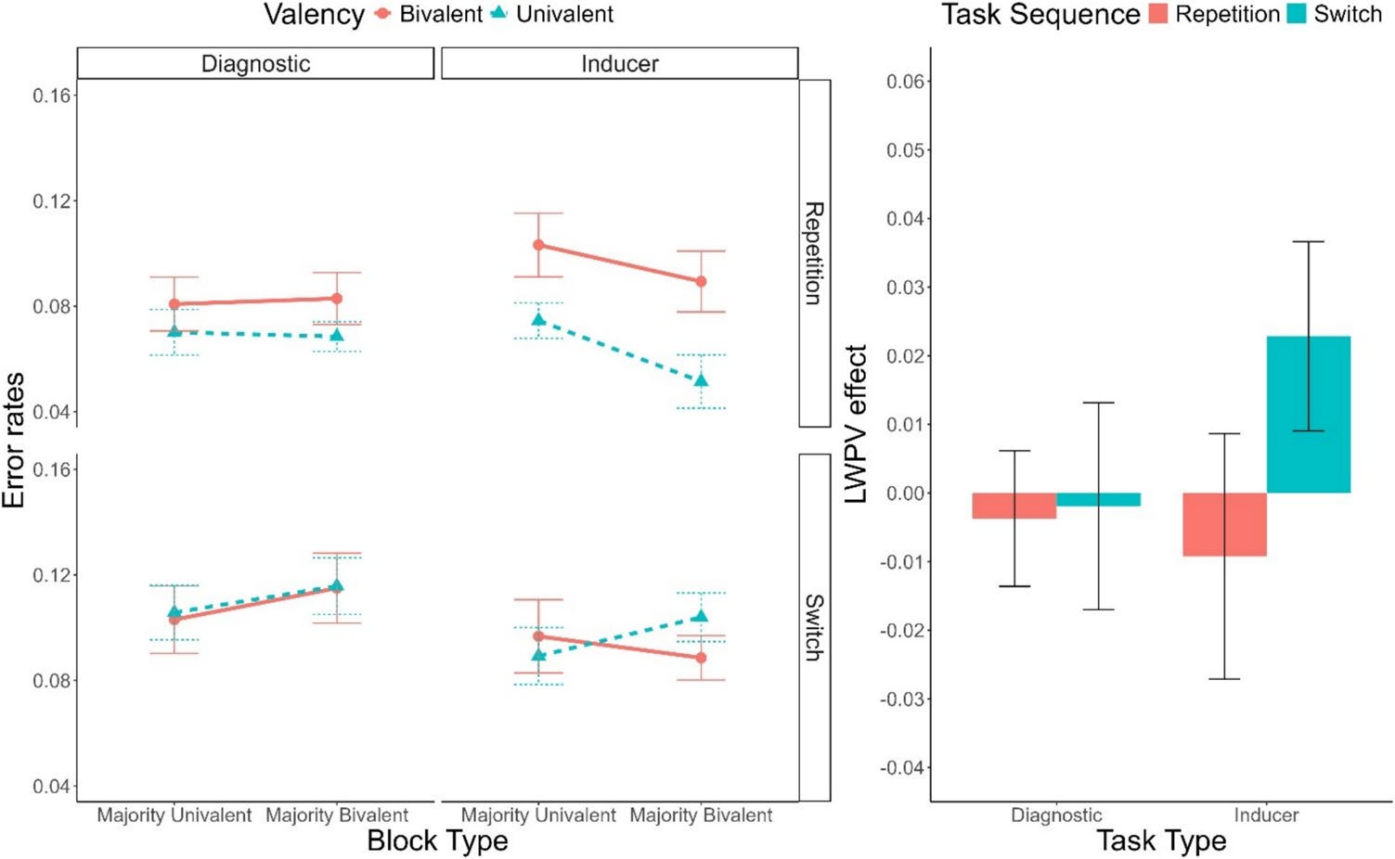
**Left panel:** Error rates as a function of Task Type (Inducer, Diagnostic), Task Sequence (Repetition, Switch), Valency (Bivalent, Univalent), and Block Type (Majority-Univalent, Majority-Bivalent). **Right panel:** List-wide proportion of valency (LWPV) effect as a function of Task Sequence and Task Type. Error bars represent the standard error of the mean

Descriptive statistics of the error rates are depicted in Fig. 4. A main effect of Task Sequence indicated the presence of significant switch cost, *F*(1, 53) = 34.43, *p* <.001, ${\upeta }_{p}^{2}$ =.39, ${\upeta }_{G}^{2}$=.03. Similarly to the RT analysis, this effect interacted with Valency, *F*(1, 53) = 13.25, *p* <.001, ${\upeta }_{p}^{2}$ =.20, ${\upeta }_{G}^{2}$ =.01, with valency effects being only present in repetition trials (2.4%), but not in switch trials (−0.2%). Again similarly to RTs, this effect was further modulated by Task Type, *F*(1, 53) = 4.52, *p* =.038, ${\upeta }_{p}^{2}$ =.08, ${\upeta }_{G}^{2}$ <.01, being restricted to the diagnostic task. Furthermore, we found a three-way interaction involving Task Sequence, Block Type, and Task Type, *F*(1, 53) = 8.05, *p* =.006, ${\upeta }_{p}^{2}$ =.13, ${\upeta }_{G}^{2}$ =.01. This interaction indicated that, in the inducer task, the switch cost was increased in majority-bivalent blocks (2.9%) compared to majority-univalent blocks (0.3%), *F*(1, 53) = 13.10, *p* <.001, ${\upeta }_{p}^{2}$ =.20, ${\upeta }_{G}^{2}$ =.04, BF_10_ = 149.98. No such effect emerged in the diagnostic task, *F*(1, 53) < 1, BF_10_ = 0.15.

### Discussion

In Experiment 2a we aimed to test whether task-conflict control was modulated in a sustained fashion depending on the block type. If this was the case, the list-wide PV effect should not depend on the identity of the currently relevant task, which can change on a trial-by-trial basis. Contrary to this idea, the frequentist analyses revealed that the list-wide PV effect was only present in the inducer task, whereas it was reversed in the diagnostic task (but notice that the Bayesian analyses did not provide support for either of these effects). Although we did not expect such a reversed pattern, we believe that this result is particularly informative of the mechansisms responsible for task-conflict control modulations. At this point, it is particularly interesting to observe that in the inducer task the list-wide PV effect was primarily driven by repetition trials. On the contrary, in the diagnostic task the reversed list-wide PV effect was only found in switch trials. We suggest that this finding may indicate a carryover of control settings from the previous trial, as detailed below.

To understand this point, it is important to consider how the control settings recruited in the inducer or the diagnostic task affect performance in the subsequent trial, depending on whether the task repeats (i.e., inducer-inducer or diagnostic-diagnostic sequences) or switches (i.e., inducer-diagnostic or diagnostic-inducer sequences). When performing the inducer task in majority-bivalent blocks, strong inhibition should be exerted on the task-irrelevant feature. If the inducer task repeats in the next trial, such inhibition of the task-irrelevant feature may carry over to the next trial. This would facilitate performance in bivalent trials, whereas it would produce no effects in univalent trials, thus generating the list-wide PV effect. If instead a switch to the diagnostic task is required, the carryover inhibition of the previously irrelevant (but currently relevant) feature should hinder performance in bivalent trials, where the two stimulus’ features compete for selection. As a result, the list-wide PV effect is reversed in the diagnostic task if the inducer task was performed in trial N-1, namely in switch trials. Complementarily, when the diagnostic task is performed in trial N-1, the effects of proportion valency on inhibition should be weaker, so that the task-irrelevant feature is inhibited only mildly in majority-bivalent blocks. As a consequence, no list-wide PV effect emerges in repetition trials. The weak inhibition of the task-irrelevant feature in the diagnostic task, however, does not impede the emergence of a list-wide PV effect when switching away from it (i.e., when switching to the inducer task).

Although speculative, this account would suggest that task-conflict control is adapted on a local basis (i.e., transiently) rather than globally. However, before drawing firm conclusions on this idea we felt the need to replicate the pattern of results observed in Experiment 2a. This choice was motivated by the unexpected observation that the list-wide PV effect was reversed in the diagnostic task, which we did not predict a priori. As highlighted above, it is particularly interesting to observe that this pattern emerges only in switch trials, whereas in the inducer task the list-wide PV effect is stronger in repetition trials in Experiment 2a. Furthermore, the results of the Bayesian analyses were inconclusive with regard to whether the list-wide PV effect emerged in the inducer task, and whether it was reversed in the diagnostic task. Given the novelty of these results, and the inconstistencies between statistical methods, Experiment 2b was run as a direct replication of Experiment 2a.

## Experiment 2b

Experiment 2b was a direct replication of Experiment 2a, and was pre-registered at: https://aspredicted.org/jqqb-pv6f.pdf. The primary aims of Experiment 2b were to: (a) replicate the reversed list-wide PV effect for the diagnostic task, and (b) confirm that such an effect emerged in switch trials (contrary to the list-wide PV effect in the inducer task which emerged more strongly in repetition trials). Sixty new participants (mean age in years = 22.3, 74.2% females) were recruited partly among students of RWTH Aachen (38) and partly on Prolific (22) in exchange for course credit or monetary compensation (10€/h).

### Results

#### Data trimming

Following the same criteria of Experiment 2a, we excluded four participants due to excessive error rate (i.e., above 33%) and five participants due to an insufficient number of trials in at least one cell of our ANOVA design (i.e., less than ten trials). After data trimming, 31.1% of trials of the initial sample were retained (notice that 55% were excluded by design, as highlighted for Experiment 2a). The mean number of trials in a cell of the design per participant was 39.9 (SD = 30.2).

#### RT analyses

**Fig. 5:**
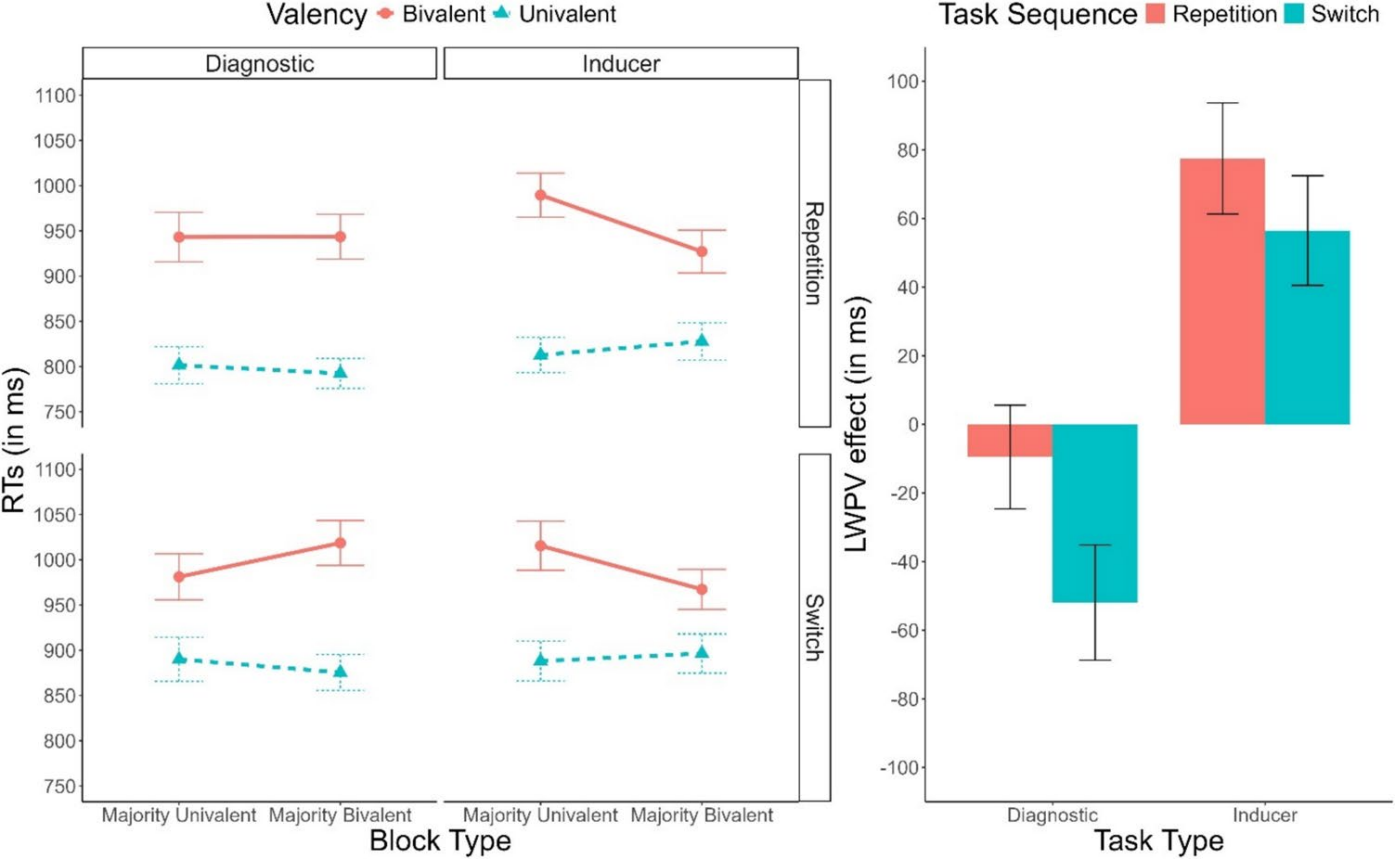
**Left panel:** Mean response times (RTs) as a function of Task Type (Inducer, Diagnostic), Task Sequence (Repetition, Switch), Valency (Bivalent, Univalent), and Block Type (Majority-Univalent, Majority-Bivalent). **Right panel:** List-wide proportion of valency (LWPV) effect as a function of Task Sequence and Task Type. Error bars represent the standard error of the mean

Descriptive statistics of the RTs are depicted in Fig. 5. The overall pattern of results was fairly similar to Experiment 2a. We observed both significant switch cost and valency effect as indicated by the main effects of Task Sequence, *F*(1, 50) = 102.86, *p* <.001, ${\upeta }_{p}^{2}$ =.67, ${\upeta }_{G}^{2}$ =.04, and Valency, *F*(1, 50) = 250.86, *p* <.001, ${\upeta }_{p}^{2}$ =.83, ${\upeta }_{G}^{2}$ =.13, respectively. These two factors interacted, *F*(1, 50) = 20.37, *p* <.001, ${\upeta }_{p}^{2}$ =.29, ${\upeta }_{G}^{2}$ <.01, replicating the result of a larger valency effect in repetition trials (142 ms) compared to switch trials (109 ms). In turn, this interaction was modulated by Block Type, *F*(1, 50) = 4.64, *p* =.036, ${\upeta }_{p}^{2}$ =.08, ${\upeta }_{G}^{2}$ <.01, indicating that while a reversed list-wide PV effect emerged in repetition trials, *F*(1, 50) = 7.54, *p* =.008, ${\upeta }_{p}^{2}$ =.13, ${\upeta }_{G}^{2}$ <.01, BF_10_ = 0.59, no list-wide PV effect was present in switch trials, *F*(1, 50) < 1, BF_10_ = 0.15. Finally, and most crucially, the three-way interaction between Valency, Block Type, and Task Type was again significant, *F*(1, 50) = 54.87, *p* <.001, ${\upeta }_{p}^{2}$ =.52, ${\upeta }_{G}^{2}$ <.01. As in Experiment 2a, a robust list-wide PV effect was observed in the inducer task, *F*(1, 50) = 31.27, *p* <.001, ${\upeta }_{p}^{2}$ =.38, ${\upeta }_{G}^{2}$ =.01, this time being also supported by the Bayesian ANOVA (BF_10_ = 17.32). More crucially, we replicated a reversed list-wide PV effect in the diagnostic task, *F*(1, 50) = 5.89, *p* =.019, ${\upeta }_{p}^{2}$ =.11, ${\upeta }_{G}^{2}$ <.01, although, again, this effect was lacking Bayesian evidence (BF_10_ = 0.45).

#### Error rate analyses

**Fig. 6:**
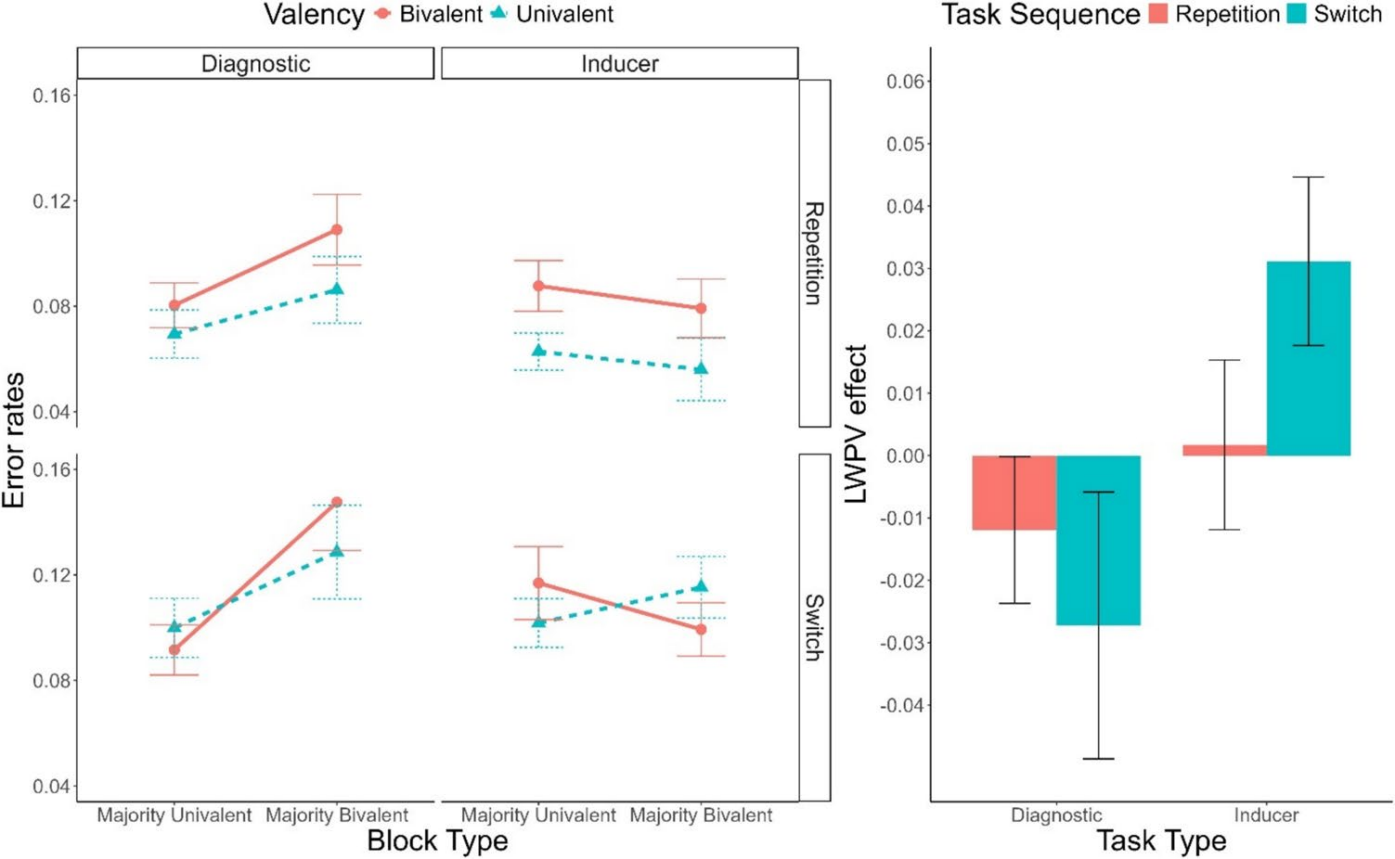
**Left panel:** Error rates as a function of Task Type (Inducer, Diagnostic), Task Sequence (Repetition, Switch), Valency (Bivalent, Univalent), and Block Type (Majority-Univalent, Majority-Bivalent). **Right panel:** List-wide proportion of valency (LWPV) effect as a function of Task Sequence and Task Type. Error bars represent the standard error of the mean

Descriptive statistics of the error rates are reported in Fig. 6. As in the RT analyses, the main effect of Task Sequence, *F*(1, 50) = 53.17, *p* <.001, ${\upeta }_{p}^{2}$ =.52, ${\upeta }_{G}^{2}$ =.04, was further modulated by Valency, *F*(1, 50) = 13.10, *p* <.001, ${\upeta }_{p}^{2}$ =.21, ${\upeta }_{G}^{2}$ <.01, indicating that the valency effect only emerged in repetition trials (2.0%), not in switch trials (0.2%). In addition, Task Sequence interacted with Block Type, *F*(1, 50) = 4.76, *p* =.034, ${\upeta }_{p}^{2}$ =.09, ${\upeta }_{G}^{2}$ <.01, revealing that the switch cost was larger in majority-bivalent blocks (4.0%) compared to majority-univalent blocks (0.2%). Finally, we found a main effect of Task Type, *F*(1, 50) = 6.90, *p* =.011, ${\upeta }_{p}^{2}$ =.12, ${\upeta }_{G}^{2}$ <.01, as more errors were made on the diagnostic task (10.2%) compared to the inducer task (9.0%). This effect was further modulated by Block Type, *F*(1, 50) = 24.38, *p* <.001, ${\upeta }_{p}^{2}$ =.33, ${\upeta }_{G}^{2}$ =.01, indicating that better performance in the diagnostic task was limited to the majority-bivalent blocks.

### Discussion

Experiment 2b replicated the most crucial results of Experiment 2a. In particular, we again observed a three-way interaction between Valency, Block Type, and Task Type, indicating a robust list-wide PV effect in the inducer task, and a reversed list-wide PV effect in the diagnostic task (although the latter did not receive support in the Bayesian analyses). Importantly, the reversed list-wide PV effect in the diagnostic task was again driven by performance in switch trials, whereas the list-wide PV effect in the inducer task was stronger in repetition trials. As discussed above, these findings suggest that the control settings employed in one trial were carried over to the next one. Under these circumstances, the reversed list-wide PV effect may result from the carryover inhibition of the previously irrelevant task feature in majority-bivalent blocks, which then becomes relevant in switch trials. On the contrary, when the task repeats, such carryover inhibition results in a smaller valency effect as observed in the diagnostic task.

Taken together the results of Experiments 2a and 2b provide evidence for a transient control-adaptation account of the list-wide PV effect, where control settings are not adapted globally throughout the block, but rather on a trial-by-trial basis. These results seemingly run counter to the findings of Experiment 1a, where we ruled out a transient control-adaptation account of the list-wide PV effect based on task-set preparation. Together, these results may suggest that, although task-conflict control is regulated in a transient fashion (as suggested by Experiments 2a and 2b), the mechanisms being regulated are not operating before stimulus onset (as suggested by Experiment 1a).

## General discussion

It is often thought that control mechanisms, including those dealing with stimulus-based task conflict (i.e., task-conflict control), can be up- or down-regulated depending on the likelihood of conflict occurrence. In paradigms such as the Stroop, it is assumed that when stimulus-based task conflict is likely to occur, participants engage in sustained inhibition of the task-irrelevant feature (Goldfarb & Henik, 2007) in order to minimize the detrimental effects that this exerts on task selection. Empirically, this modulation of control is demonstrated by the list-wide PV effect, which is a robust finding in the Stroop task (Entel & Tzelgov, 2018; Entel et al., 2015; Goldfarb & Henik, 2007; Kalanthroff et al., 2013; Keha & Kalanthroff, 2023a, 2023b), and was recently replicated by us in task-switching (Moretti et al., 2025). Although it may be tempting to conclude that the same adaptation mechanisms are behind the list-wide PV effect in both paradigms, we reasoned that sustained inhibition of the task-irrelevant feature is not an available strategy in a task-switching paradigm. Therefore, the present study sought to answer an open question: How is task-conflict control adapted in task switching?

In Experiments 1a and 1b (reported in the Appendix), we tested the idea that task-conflict control may be upregulated transiently on a trial-by-trial basis. In particular, we hypothesized that cue-based preparation may be modulated as a function of conflict probability, with stronger preparation being accomplished in majority-bivalent blocks. In fact, it is often hypothesized that an important component of advance reconfiguration is attentional biasing (Logan & Gordon, 2001; Longman et al., 2013; Meiran, 2000). According to this idea, participants are able to prepare to direct their attention to the task-relevant feature during the CSI. Although some residual carryover of previous attentional settings remains (Elchlepp et al., 2017; Longman et al., 2014), attentional biasing has the potential to strongly reduce stimulus-based task conflict. Therefore, modulating the strength, or the probability to engage (De Jong, 2000), in cue-based preparation across block types has the potential to produce the list-wide PV effect. Contrary to our predictions, however, CSI manipulations did not impact task-conflict control.

In Experiment 1b, where the CSI was manipulated between subjects, we could not establish that cue-based preparation was stronger in the long-CSI group. As this was a pre-requisite for testing whether preparation impacts the list-wide PV effect, we decided to report this experiment in the Appendix, where we also discuss the limits of manipulating the CSI between subjects. In order to remediate this issue, in Experiment 1 the CSI varied unpredictably within subjects. In line with previous literature (Altmann, 2004; Koch, 2001), these efforts were successful in generating cue-based preparation effects. Nonetheless, CSI did not modulate the list-wide PV effect. These results suggest that a difference in attentional biasing during the CSI is not the mechanism responsible for the emergence of the list-wide PV effect.

Moving on from these results, in Experiments 2a and 2b we aimed to test whether task-conflict control is modulated in a sustained fashion. Borrowing from the proportion-switch literature (Dreisbach & Fröber, 2019), we tested the idea that frequent task conflict may call for increased task shielding, possibly implemented by decreasing the activation of task-sets into working memory. If this, or any kind of sustained process, is responsible for the occurrence of the list-wide PV effect, we should observe this effect to generalize across any feature changing on a trial-by-trial basis (cf. Braem et al., 2019). The results of Experiments 2a and 2b run opposite to this prediction. We observed a significant list-wide PV effect only in the task that was mostly associated with bivalent trials (inducer task), and not in another task whose proportion of bivalent trials was kept constant across blocks (diagnostic task). This interaction demonstrates that sustained processes operating uniformly throughout a block cannot be responsible for generating the list-wide PV effect, as this appears to be task-specific (cf. Siqi-Liu & Egner, 2020).

In the diagnostic task we even found the valency effect to increase in majority-bivalent blocks. Although this effect did not receive support in the Bayesian analyses, suggesting that some caution is needed, we interpret such a reversed list-wide PV effect as evidence for adaptation mechanisms to operate transiently. In this regard, it is interesting to note that the reversed list-wide PV effect was only present in switch trials, whereas the regular list-wide PV effect observed in the inducer task was driven by repetition trials. We interpret these findings to indicate that carryover effects of control states severely impacted the emergence of list-wide PV effect in Experiments 2a and 2b. In particular, switching away from the inducer task in majority-bivalent blocks results in increased task conflict, as the now-relevant stimulus feature was strongly inhibited in the previous trial. On the contrary, in majority-univalent blocks, switching away from the inducer task does not impact performance as much since the now-relevant stimulus feature was not strongly inhibited in the previous trial. To summarize, the results from Experiments 2a and 2b suggest that transient control mechanisms, operating on a time scale of a few trials, are responsible for the emergence of the list-wide PV effect. Considering the results of Experiment 1a, however, our conclusion is that these transient mechanisms do not target cue-based preparation, but possibly some later processes that follow stimulus onset.

### Alternative mechanisms behind the list-wide PV effect.

The question therefore remains: How is control upregulated to deal with frequent stimulus-based task conflict? One first conclusion can be drawn comparing the results of Experiment 1a with those of Experiments 2a and 2b. One crucial difference among these experiments is that in Experiments 2a and 2b the proportion of bivalent trials was manipulated in a task-specific fashion, so that participants could make use of task-control associations to guide performance. In these experiments we found a stronger list-wide PV effect in repetition trials for the inducer task, whereas this effect was reversed in switch trials for the diagnostic task. As mentioned above, the pattern observed in Experiments 2a and 2b is suggestive of task-specific control settings that carry over to the next trial. When performing the inducer task, strong inhibition of the task-irrelevant feature in majority-bivalent blocks diminishes task conflict in repetition trials (thus producing the list-wide PV effect for the inducer task), but increases it in switch trials (thus producing the reversed list-wide PV effect for the diagnostic task). If similar mechanisms were at stake when the proportion of bivalent trials was manipulated for both tasks, we should have observed the same pattern of results (i.e., stronger list-wide PV effect in repetition trials) both in Experiments 1a and 1b and in our previous study (Moretti et al., 2025, Experiment 2). On the contrary, however, in those experiments the list-wide PV effect was strongest in switch trials. This comparison suggests that introducing task-control associations changes the nature of conflict adaptation radically.

At this point, we can only speculate on the reasons why introducing task-control associations may change the strategy employed by the participants, inducing control states to carryover from one trial to another. One possibility is suggested by the Stroop literature, where the effects of introducing inducer and diagnostic items have been investigated extensively (Blais & Bunge, 2010; Blais et al., 2007; Braem et al., 2019; Bugg et al., 2008). These investigations have been conducted in the context of the list-wide proportion congruency (PC) effect, namely the observation that the congruency effect, measuring stimulus-based response conflict, is reduced when incongruent trials are frequent (Bugg & Chanani, 2011; Bugg & Crump, 2012; Spinelli & Lupker, 2023; Spinelli et al., 2019; Torres-Quesada et al., 2013, 2014). In this literature, it has been found that the list-wide PC effect often does not generalize to diagnostic items, inspiring a debate about whether this effect actually reflects sustained top-down biasing of attention, or rather some bottom-up mechanism triggered by stimulus-control associations (Blais & Bunge, 2010; Blais et al., 2007; Braem et al., 2019; Bugg et al., 2008). One important step forward in this debate was made by Bugg (2014), who showed that the list-wide PC effect did not transfer to diagnostic items when reliable stimulus–response associations were available to the participants, but did when such associations were absent. The conclusion that can be drawn from this literature is that when participants are given the option to use contingencies, or stable item/location/task-control associations, they tend to do so (cf. Verguts & Notebaert, 2009; Xu et al., 2024). Similarly, our study suggests that when participants can use task-control associations, the mechanisms regulating task-conflict control change substantially, so that local effects such as carryover of control settings play a crucial role.

In light of this finding, we should still ask ourselves how control is regulated when task-control associations are not available. Although the results of Experiments 2a and 2b are not in line with sustained control adaptation, Experiment 1b failed to confirm that control is regulated transiently through a strengthening of cue-based preparation. In this regard, we can think of at least two alternative possibilities. First, it is possible that the likelihood of stimulus-based task conflict influences processes taking place after stimulus onset. This idea traces back to the notion that attentional biasing can only be achieved to some degree following cue onset, with the rest of the work being completed only when the stimulus is presented (Elchlepp et al., 2017; Longman et al., 2014; Meiran, 2000; Rogers & Monsell, 1995), causing the emergence of a residual switch cost even with long preparation time. In our paradigm, where cue-based preparation is attenuated due to the small percentage of incongruent trials, the post-stimulus component may be more relevant in determining the size of the valency effect. Although speculative, this idea is supported by the finding that valency did not interact significantly with CSI. Therefore, it is possible that in majority-bivalent blocks, reactive control mechanisms are more finely tuned to deal with the emergence of task conflict (cf. Wendt et al., 2013). This idea would suggest that the list-wide PV effect does not emerge from proactive mechanisms, but rather from reactive ones. Although, as mentioned above, in the Stroop literature the list-wide PC effect emerges even when controlling for a reactive component (Bugg, 2014; Spinelli et al., 2019), the results from Experiment 1 seem to suggest that this may not be the case in task switching (cf. Siqi-Liu & Egner, 2020, 2023).

A second possibility is that attentional biasing is successfully achieved within 200 ms in our paradigm. This idea is also consistent with a lack of interaction between CSI and Valency. If attentional biasing was not completed in 200 ms, it is reasonable to assume that giving more time to prepare should reduce the valency effect, directing participants’ attention toward the task-relevant feature in bivalent trials. However, this idea would run counter to previous attention-switching literature, in which the CSI was found to modulate attentional biasing. Using eye tracking, Longman and collaborators (2013; Longman et al., 2014, 2016) were able to show that fixations to the previously relevant stimulus dimension were reduced as a function of the CSI (although never completely eliminated). Based on these findings, we therefore deem unlikely the possibility that attentional biasing would be completed within 200 ms.

### Relation to the stability-flexibility tradeoff debate

Although not the focus of the present study, we believe that some of our results are particularly interesting in the light of a recent debate about the existence of a tradeoff between cognitive stability and flexibility. As mentioned in the *Introduction*, it has been proposed that cognitive flexibility trades off against the ability to shield a task-set from interference (i.e., stability). For instance, Dreisbach and Fröber (2019) assume that increasing the proportion of switches in a block results in stronger activation of all task-sets, thus reducing the switch cost. At the same time, however, increasing task-set activation should also result in greater task conflict. Therefore, this account implicitly predicts that stability and flexibility necessarily trade off. In a recent study, Geddert and Egner (2022) empirically tested this prediction by manipulating both the proportion of switch and incongruent trials across blocks. Taking the switch cost as a measure of cognitive flexibility, and the congruency effect as a measure of task conflict, it would be expected that increasing the proportion of switch trials results in a larger congruency effect (in addition to smaller switch cost). Complementarily, if increasing the proportion of incongruent trials results in greater task shielding, the switch cost should be larger in majority-incongruent blocks (in addition to the smaller congruency effect). Contrary to these predictions, the switch cost was not affected by congruency proportion, and, complementarily, the congruency effect did not vary as a function of switch proportion. The authors concluded that cognitive flexibility and stability are not two ends of a continuum, but rather two separate dimensions, so that a tradeoff between these two is not necessary (see also Geddert & Egner, 2024).

However, it is important to notice that the congruency effect may not be an ideal measure of task conflict. As mentioned in the *Introduction*, in the Stroop literature the congruency effect is generally thought to reflect conflict in response selection rather than task conflict (Augustinova et al., 2019; MacLeod, 1991). A similar distinction was also proposed in task switching, where stimuli are thought to automatically trigger the stimulus–response mapping of both tasks via a direct route (Kiesel et al., 2007; Proctor et al., 2011; Wendt & Kiesel, 2008), thus causing response conflict in incongruent trials (however, see Meiran & Kessler, 2008; Schneider, 2015). For these reasons, we argue that the operationalization of task conflict may be imprecise in the study of Geddert and Egner (2022), making their results difficult to interpret.

In the present study we opted for what we believe to be a better solution to measuring task conflict, operationalizing it as the difference in performance between congruent and univalent trials, two conditions where response conflict is absent altogether. Although not the focus of the present study, this set-up allows to establish whether increasing the frequency of task conflict trials (i.e., bivalent trials) impacts cognitive flexibility as measured with the switch cost. In line with a tradeoff between stability and flexibility, we report a larger switch cost in majority-bivalent blocks for all datasets. In other words, a better capacity to shield the currently relevant task from stimulus-based task conflict (as signaled by a smaller valency effect) comes at the expense of reduced cognitive flexibility (as indicated by a larger switch cost). Although our results are not necessarily incompatible with Geddert and Egner (2022, 2024), who proposed that stability and flexibility do not *always* trade off (rather than suggesting that they *never* do), we believe that they substantiate an important concern over the operationalization of task conflict in their study. Indeed, applying a similar logic, but manipulating task conflict with the proportion of bivalent trials, our results run opposite to theirs.

### Conclusions

The aim of the present study was to shed light on how task-control is adapted across different environments in task switching. Two conclusions can be drawn from our findings. The first is that control adaptation in task switching is preferably achieved by making use of task-control associations when these are available (cf. Siqi-Liu & Egner, 2020). This finding contrasts with a sustained control-adaptation account positing that control adaptation is driven by mechanisms acting uniformly across all trials of a block. The second conclusion that can be drawn is that task-control adaptation in task switching is not achieved by modulating attentional biasing before stimulus onset. We propose instead that task-conflict control is likely adapted by modulating processes that take place after stimulus onset.

## Electronic supplementary material

Below is the link to the electronic supplementary material.

Supplementary file1 (DOCX 643 KB)

## Figures and Tables

**Fig. 7:**
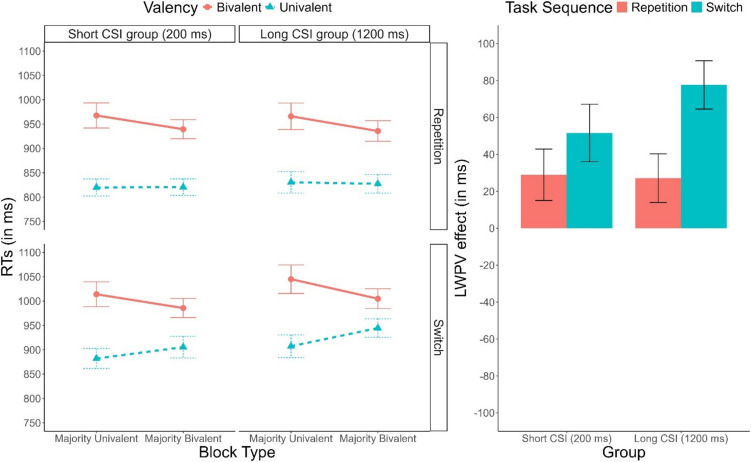
**Left panel:** Mean response time (RT) as a function of CSI (Short, Long), Task Sequence (Repetition, Switch), Valency (Bivalent, Univalent) and Block Type (Majority-Univalent, Majority-Bivalent). **Right panel:** Mean list-wide proportion of valency effect as a function of Task Sequence and CSI. Error bars represent the standard error of the mean

**Fig. 8:**
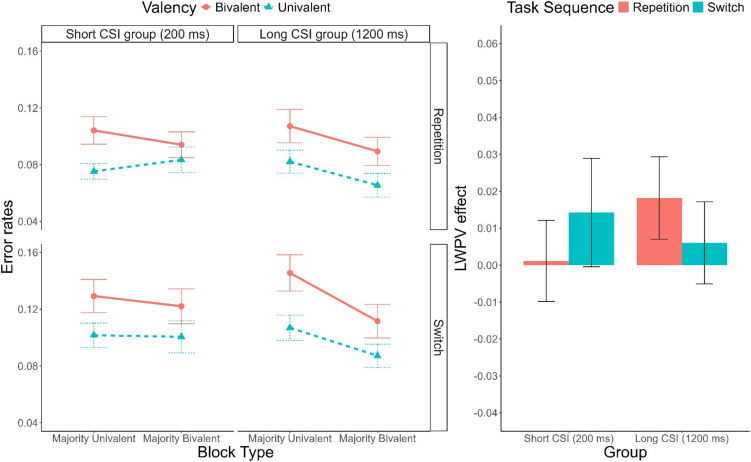
**Left panel:** Mean error rate as a function of CSI (Short, Long), Task Sequence Repetition, Switch), Valency (Bivalent, Univalent) and Block Type (Majority-Univalent, Majority-Bivalent). **Right panel:** Mean list-wide proportion of valency effect as a function of Task Sequence and CSI. Error bars represent the standard error of the mean

## Data Availability

Raw data and analyses scripts are available at: https://pasa.psycharchives.org/reviewonly/e011f0b171932e101801d3c568f242304326464f8b463583dea61691dd0ffd6f.

## Appendix

In the Appendix we report the methods and results of Experiment 1b. The aim of Experiment 1b was the same as Experiment 1a reported in the main text. Specifically, we intended to test whether the list-wide PV effect emerges from stronger task-set preparation in majority-bivalent blocks during the CSI. However, as opposed to Experiment 1a, the CSI was manipulated between subjects. This choice was made as previous studies found differential effects of cue-based preparation when using between- and within-subjects manipulations, with effects being stronger in within-subjects designs (Altmann, 2004; Koch, 2001).

## Methods

### Participants

Experiment 1b was pre-registered at https://aspredicted.org/ji5yi.pdf. 120 participants took part in the experiment (mean age in years = 23.5, 60.9% females) in exchange for partial-course credits or monetary compensation (20€). The sample size was determined similarly to what pointed out for Experiment 1a, except that instead of recruiting 60 participants in total, we recruited 60 participants per group. Half of the sample was assigned to the short CSI condition, and the other half was assigned to the long CSI condition. Two participants, one for each group, were excluded due to technical issues, although this criterion was not pre-registered. In particular, for those participants the task cue was displayed for at least 20 ms longer/shorter in over 10% of the trials. Following data trimming (see below), the sample included 105 participants, 54 in the long CSI group and 51 in the short CSI group. The experiment was built and run online using Gorilla (Anwyl-Irvine et al., 2020). Part of the sample (24 participants in each group) were recruited through Prolific.

## Methods and statistical design

The experiment was identical to Experiment 1a in every respect except for the between-subjects manipulation of CSI. This ensured that we had more trials in each condition of the statistical design. Separate 2 × 2 × 2 × 2 mixed ANOVAs were conducted on RTs and arcsine-transformed error rates (Laurencelle & Cousineau, 2023). In both ANOVAs the within-subjects independent variables were Valency (Bivalent, Univalent), Block Type (Majority-bivalent, Majority-univalent), and Task Sequence (Repetition, Switch). CSI (Long, Short) was manipulated between subjects.

## Results

### Data trimming

Data trimming was identical to that reported for Experiment 1a except that, following trial exclusion, we excluded participants with less than 15 trials in any cell of the statistical design (two participants). In addition, eight participants were excluded due to excessive error rate (i.e., > 33% errors), and two participants for providing suspiciously fast responses (RT < 200 ms) in more than 10% of the trials. At the end of data trimming we retained 32.6% of the trials for the included participants. The mean number of survived trials in each cell of the design per participant was 83.5 (SD = 87.6). Note that excluding both incongruent and N-1 bivalent trials led to an exclusion of 59.4% of all trials by design.

## RT analyses

Descriptive statistics of the RTs are depicted in Fig. 7. As expected, we found a significant switch cost, *F*(1, 103) = 261.76, *p* <.001, ${\upeta }_{p}^{2}$ =.72, ${\upeta }_{G}^{2}$ =.05, and valency effect, *F*(1, 103) = 319.04, *p* <.001, ${\upeta }_{p}^{2}$ =.76, ${\upeta }_{G}^{2}$ =.12. Surprisingly, however, the switch cost was significantly larger in the long CSI group (86 ms) compared to the short CSI group (60 ms), as indicated by the interaction between Group and Task Sequence, *F*(1, 103) = 8.17, *p* =.005, ${\upeta }_{p}^{2}$ =.07, ${\upeta }_{G}^{2}$ <.01. Valency interacted with Task Sequence, *F*(1, 103) = 17.49, *p* <.001, ${\upeta }_{p}^{2}$ =.15, ${\upeta }_{G}^{2}$ =.01, indicating a larger valency effect in repetition trials (128 ms) than in switch trials (102 ms). Furthermore, Valency also interacted with Block Type, *F*(1, 103) = 34.39, *p* <.001, ${\upeta }_{p}^{2}$ =.25, ${\upeta }_{G}^{2}$ =.01, revealing a significant list-wide PV effect with a smaller valency effect in majority-bivalent blocks (92 ms) compared to majority-univalent blocks (137 ms). This interaction was further modulated by Task Sequence, *F*(1, 103) = 9.01, *p* =.003, ${\upeta }_{p}^{2}$ =.08, ${\upeta }_{G}^{2}$ <.01. Replicating results from our previous study (Moretti et al., 2025), the list-wide PV effect was indeed larger in switch trials (63 ms), *F*(1, 104) = 39.30, *p* <.001, ${\upeta }_{p}^{2}$ =.27, ${\upeta }_{G}^{2}$ =.01, BF_10_ = 17.7, than in repetition trials (27 ms), *F*(1, 104) = 8.64, *p* =.004, ${\upeta }_{p}^{2}$ =.08, ${\upeta }_{G}^{2}$ <.01, BF_10_ = 0.5. Finally, Task Sequence interacted with Block Type, *F*(1, 103) = 3.96, *p* =.049, ${\upeta }_{p}^{2}$ =.04, ${\upeta }_{G}^{2}$ <.01, revealing a larger switch cost in majority-bivalent blocks (79 ms) compared to majority-univalent blocks (66 ms).

## Error rate analyses

Descriptive statistics of the error rates are depicted in Fig. 8. The only significant effects were those of Task Sequence, *F*(1, 103) = 37.37, *p* <.001, ${\upeta }_{p}^{2}$ =.27, ${\upeta }_{G}^{2}$ =.03, indicating the emergence of the switch cost, Valency, *F*(1, 103) = 40.88, *p* <.001, ${\upeta }_{p}^{2}$ =.28, ${\upeta }_{G}^{2}$ =.02, indicating a significant valency effect, and Block Type, *F*(1, 103) = 10.75, *p* =.001, ${\upeta }_{p}^{2}$=.09, ${\upeta }_{G}^{2}$ =.01, indicating that performance was overall worse in majority-univalent blocks compared to majority-bivalent blocks.

## Discussion

In Experiment 1b we tested the hypothesis that task-conflict control would be upregulated in majority-bivalent blocks through a strengthening of cue-based preparation. We therefore manipulated the CSI between participants in order to allow cue-based preparation to take place in one group (long CSI), but not in the other group (short CSI). If stronger cue-based preparation in majority-bivalent blocks is responsible for the list-wide PV effect, we should find a decreased or even abolished list-wide PV effect when participants had little time to prepare. Surprisingly, however, we could not find cue-based preparation effects in the first place. In fact, not only did performance not improve overall in the long CSI group, but the switch cost was even larger compared to the short CSI group. We attribute this surprising result to two factors.

Generally speaking, between-subjects manipulations of the CSI are often found to impact the switch cost to a lesser degree compared to within-subjects manipulations, with some studies finding no modulation at all (e.g., Altmann, 2004; Koch, 2001; Koch & Allport, 2006). This factor alone, however, does not fully clarify why the switch cost could be reversed. The second factor to consider, which differentiates ours from previous studies, is that in a large proportion of trials maintaining cue information was not relevant to correctly performing the task at hand, especially in majority-univalent blocks. In this block type, 75% of the trials were univalent, and 12.5% were congruent. As a result, in 87.5% of the trials participants did not need to process the cue to correctly perform the task. In majority-bivalent blocks, 25% of the trials were univalent and 37.5% were congruent, so that participants did not need to process the cue in 62.5% of the trials. Under these circumstances, a long CSI is detrimental to performance as the information provided by the cue should be kept longer in working memory. While in repetition trials this effect is partially counteracted by the carryover activation of the previously relevant task, performance in switch trials is impaired to a higher degree. In this regard, we could consider cue presentation as a warning signal, and the observed increase in switch cost as a fixed fore-period effect, namely the increase in RTs at long CSIs when the CSI is blocked (Los & Van Den Heuvel, 2001).

In order to remediate these issues, in Experiment 1a we manipulated the CSI within subjects and we avoided blocking the CSI, making it unpredictable. These efforts were successful in generating cue-based preparation effects. Interestingly, we found some evidence that cue-based preparation occurred uniquely in majority-bivalent blocks, consistent with the idea that in majority-univalent blocks cue information is mostly not helpful in performing the correct task. Nonetheless, CSI did not modulate the list-wide PV effect. Taken together, the results from Experiments 1a and 1b suggest that a difference in attentional biasing during the CSI is not the responsible mechanism for the emergence of the list-wide PV effect.

## Footnotes

1 Note that the same mechanisms are also often invoked to account for other proportion effects in the Stroop paradigm, such as the list-wide proportion congruency effect, or item-specific proportion congruency effects. Here we decided to focus on the effects related to task-conflict control as these are most relevant for our study.

2 We only performed Bayesian ANOVAs when decomposing higher-order interactions, and not also when running the complete ANOVA model with all factors (i.e., the four-way ANOVA), for two reasons. First, following the reported method for Bayesian ANOVAs (i.e., building all models with a high number of iterations) these computations would be too time consuming (each four-way ANOVA would require months with our equipment). Second, and most crucially, our effect of interest (i.e., the three-way interaction between CSI, Valency, and Block Type) was not predicted to be a null effect.

## Declarations

### Open practices

Pre-registration protocols are available at: https://aspredicted.org/ZNW_J49, https://aspredicted.org/ji5yi.pdf and.https://aspredicted.org/jqqb-pv6f.pdf
