# Supplementary material for: Task-control adaptation in task switching: Uncovering the mechanisms behind the list-wide proportion valency effect
Source: Mem Cognit. 2025 Sep 15;54(3):813–34. doi: 10.3758/s13421-025-01782-1 (PMC13132911; doi:10.3758/s13421-025-01782-1)
Supplement: Supplementary file 1 — Supplementary file1 (DOCX 643 KB) [file 13421_2025_1782_MOESM1_ESM.docx]

**Online supplementary materials of “More on control adaptation in task switching: Testing a sustained and a transient account of the list-wide proportion of valency effect.”**

**Table S1:** Full table of results for the RT ANOVA in Experiment 1a.

**Table S2:** Full table of results for the error rates ANOVA in Experiment 1a.

**Table S3:** Full table of results for the RT ANOVA in Experiment 1b.

**Table S4:** Full table of results for the error rates ANOVA in Experiment 1b.

**Table S5:** Full table of results for the RT ANOVA in Experiment 2a.

**Table S6:** Full table of results for the error rates ANOVA in Experiment 2a.

**Table S7:** Full table of results for the RT ANOVA in Experiment 2b.

**Table S8:** Full table of results for the error rates ANOVA in Experiment 2b.

**Figure 1:** Depiction of the number of trials in each condition of Experiment 1a.

**Figure 2:** Depiction of the number of trials in each condition of Experiment 1b.

**Figure 3:** Depiction of the number of trials in each condition of Experiment 2a-b.

| Effect | *F* | *p* | $\eta_{p}^{2}$ |
| --- | --- | --- | --- |
| Group | 0.36 | .548 | .01 |
| **Sequence** | **261.76** | **.001** | **.72** |
| **Valency** | **319.04** | **.001** | **.76** |
| Block Type | 0.42 | .519 | .01 |
| **Group x Sequence** | **8.17** | **.005** | **.07** |
| Group x Valency | 0.52 | .473 | .01 |
| Group x Block Type | 0.00 | .973 | .01 |
| **Sequence x Valency** | **17.49** | **.001** | **.15** |
| **Sequence x Block Type** | **3.96** | **.049** | **.04** |
| **Valency x Block Type** | **34.39** | **.001** | **.25** |
| Group x Sequence x Valency | 0.13 | .717 | .01 |
| Group x Sequence x Block Type | 0.09 | .762 | .01 |
| Group x Valency x Block Type | 0.59 | .444 | .01 |
| **Sequence x Valency x Block Type** | **9.01** | **.003** | **.08** |
| Group x Sequence x Valency x Block Type | 1.34 | .250 | .01 |

***Table S1.*** Experiment 1a: Complete RTs ANOVA results. Effects reaching significance are highlighted in bold.

| Effect | *F* | *p* | $\eta_{p}^{2}$ |
| --- | --- | --- | --- |
| Group | 0.22 | .638 | .01 |
| **Sequence** | **37.37** | **.001** | **.27** |
| **Valency** | **40.88** | **.001** | **.28** |
| **BlockType** | **10.75** | **.001** | **.09** |
| Group x Sequence | 0.57 | .453 | .01 |
| Group x Valency | 0.60 | .442 | .01 |
| Group x BlockType | 2.63 | .108 | .02 |
| Sequence x Valency | 0.50 | .479 | .01 |
| Sequence x BlockType | 0.19 | .664 | .01 |
| Valency x BlockType | 0.13 | .724 | .01 |
| Group x Sequence x Valency | 0.00 | .982 | .01 |
| Group x Sequence x BlockType | 0.11 | .740 | .01 |
| Group x Valency x BlockType | 0.09 | .761 | .01 |
| Sequence x Valency x BlockType | 0.05 | .819 | .01 |
| Group x Sequence x Valency x BlockType | 1.50 | .223 | .01 |

***Table S2.*** Experiment 1a: Complete error rates ANOVA results. Effects reaching significance are highlighted in bold.

| Effect | *F* | *p* | $\eta_{p}^{2}$ |
| --- | --- | --- | --- |
| **Sequence** | **72.85** | **.001** | **.56** |
| **Valency** | **146.44** | **.001** | **.72** |
| BlockType | 0.88 | .352 | .01 |
| **CSI** | **36.31** | **.001** | **.39** |
| **Sequence x Valency** | **54.74** | **.001** | **.49** |
| Sequence x BlockType | 3.45 | .068 | .06 |
| **Valency x BlockType** | **35.40** | **.001** | **.38** |
| Sequence x CSI | 2.48 | .121 | .04 |
| Valency x CSI | 0.28 | .600 | .01 |
| BlockType x CSI | 3.37 | .072 | .05 |
| Sequence x Valency x BlockType | 0.20 | .654 | .01 |
| Sequence x Valency x CSI | 3.93 | .052 | .06 |
| Sequence x BlockType x CSI | 1.67 | .202 | .03 |
| Valency x BlockType x CSI | 0.06 | .804 | .01 |
| **Sequence x Valency x BlockType x CSI** | **6.34** | **.015** | **.10** |

***Table S3.*** Experiment 1b: Complete RTs ANOVA results. Effects reaching significance are highlighted in bold.

| Effect | *F* | *p* | $\eta_{p}^{2}$ |
| --- | --- | --- | --- |
| **Sequence** | **32.73** | **.001** | **.36** |
| Valency | 3.05 | .086 | .05 |
| **BlockType** | **4.42** | **.040** | **.07** |
| CSI | 3.12 | .083 | .05 |
| Sequence x Valency | 2.06 | .156 | .03 |
| Sequence x BlockType | 3.01 | .088 | .05 |
| Valency x BlockType | 0.21 | .648 | .01 |
| **Sequence x CSI** | **5.43** | **.023** | **.09** |
| Valency x CSI | 2.71 | .105 | .04 |
| BlockType x CSI | 0.49 | .486 | .01 |
| Sequence x Valency x BlockType | 1.69 | .199 | .03 |
| Sequence x Valency x CSI | 0.00 | .994 | .01 |
| **Sequence x BlockType x CSI** | **12.27** | **.001** | **.17** |
| Valency x BlockType x CSI | 0.31 | .579 | .01 |
| Sequence x Valency x BlockType x CSI | 0.00 | .954 | .01 |

***Table S4.*** Experiment 1b: Complete error rates ANOVA results. Effects reaching significance are highlighted in bold.

| Effect | *F* | *p* | $\eta_{p}^{2}$ |
| --- | --- | --- | --- |
| **Sequence** | **129.90** | **< .001** | **.71** |
| **Valency** | **220.50** | **< .001** | **.81** |
| Block Type | 0.04 | .839 | < .01 |
| Task Type | 0.02 | .888 | < .01 |
| **Sequence x Valency** | **12.02** | **.001** | **.18** |
| Sequence x Block Type | 1.27 | .265 | .02 |
| Valency x Block Type | 0.94 | .335 | .02 |
| **Sequence x Task Type** | **6.24** | **.016** | **.11** |
| Valency x Task Type | 0.11 | .737 | < .01 |
| **Block Type x Task Type** | **48.84** | **< .001** | **.48** |
| Sequence x Valency x Block Type | 3.34 | .073 | .06 |
| **Sequence x Valency x TaskType** | **9.07** | **.004** | **.15** |
| Sequence x Block Type x Task Type | 2.38 | .129 | .04 |
| **Valency x Block Type x Task Type** | **15.52** | **< .001** | **.23** |
| Sequence x Valency x Block Type x Task Type | 0.11 | .739 | < .01 |

***Table S5.*** Experiment 2a: complete RTs ANOVA results. Effects reaching significance are highlighted in bold.

| Effect | *F* | *p* | $\eta_{p}^{2}$ |
| --- | --- | --- | --- |
| **Sequence** | **34.43** | **<.001** | **.39** |
| Valency | 0.65 | .423 | .01 |
| BlockType | 0.28 | .602 | < .01 |
| TaskType | 3.29 | .075 | .06. |
| **Sequence x Valency** | **13.25** | **< .001** | **.20** |
| **Sequence x BlockType** | **7.51** | **.008** | **.12** |
| Valency x BlockType | 2.09 | .154 | .04 |
| Sequence x TaskType | 0.87 | .356 | .02 |
| Valency x TaskType | 2.65 | .109 | .05 |
| BlockType x TaskType | 3.55 | .065 | .06 |
| Sequence x Valency x BlockType | 1.90 | .174 | .03 |
| **Sequence x Valency x TaskType** | **4.52** | **.038** | **.08** |
| **Sequence x BlockType x TaskType** | **8.05** | **.006** | **.13** |
| Valency x BlockType x TaskType | 3.08 | .085 | .05 |
| Sequence x Valency x BlockType x TaskType | 1.67 | .202 | .03 |

***Table S6.*** Experiment 2a: Complete error rates ANOVA results. Effects reaching significance are highlighted in bold.

| Effect | *F* | *p* | $\eta_{p}^{2}$ |
| --- | --- | --- | --- |
| **Sequence** | **102.86** | **< .001** | **.67** |
| **Valency** | **250.86** | **< .001** | **.83** |
| Block Type | 0.25 | .618 | < .01 |
| Task Type | 1.05 | .310 | .02 |
| **Sequence x Valency** | **20.37** | **<.001** | **.29** |
| Sequence x Block Type | 1.45 | .235 | .03 |
| Valency x Block Type | 3.03 | .088 | .06 |
| Sequence x Task Type | 3.10 | .084 | .06 |
| Valency x Task Type | 1.45 | .234 | .03 |
| **Block Type x Task Type** | **5.28** | **.026** | **.10** |
| **Sequence x Valency x Block Type** | **4.64** | **.029** | **.08** |
| Sequence x Valency x TaskType | 0.29 | .595 | < .01 |
| Sequence x Block Type x Task Type | 0.62 | .435 | .01 |
| **Valency x Block Type x Task Type** | **54.87** | **< .001** | **.52** |
| Sequence x Valency x Block Type x Task Type | 0.57 | .455 | .01 |

***Table S7.*** Experiment 2b: complete RTs ANOVA results. Effects reaching significance are highlighted in bold.

| Effect | *F* | *p* | $\eta_{p}^{2}$ |
| --- | --- | --- | --- |
| **Sequence** | 53.17 | < .001 | .52 |
| Valency | 2.22 | .143 | .04 |
| BlockType | 0.50 | .484 | < .01 |
| **TaskType** | **6.90** | **.011** | **.12.** |
| **Sequence x Valency** | **13.10** | **< .001** | **.21** |
| **Sequence x BlockType** | **4.76** | **.034** | **.09** |
| Valency x BlockType | 3.20 | .080 | .06 |
| Sequence x TaskType | 3.21 | .079 | .06 |
| Valency x TaskType | 0.02 | .883 | < .01 |
| **BlockType x TaskType** | **24.38** | **< .001** | **.33** |
| Sequence x Valency x BlockType | 0.70 | .407 | .01 |
| Sequence x Valency x TaskType | 1.93 | .171 | .04 |
| Sequence x BlockType x TaskType | 1.18 | .283 | .02 |
| Valency x BlockType x TaskType | 0.14 | .711 | < .01 |
| Sequence x Valency x BlockType x TaskType | 1.22 | .275 | .02 |

***Table S8.*** Experiment 2b: Complete error rates ANOVA results. Effects reaching significance are highlighted in bold.


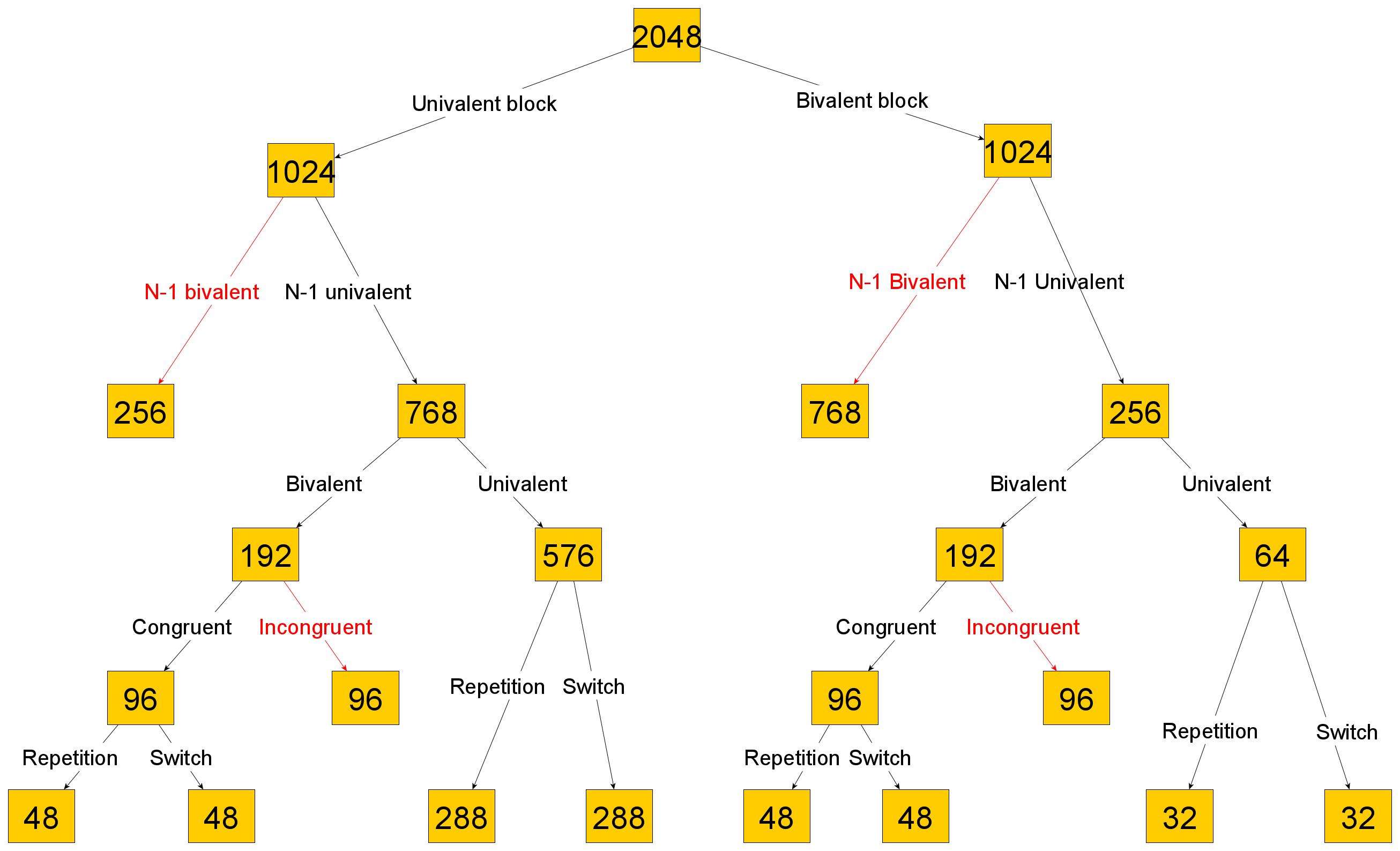


***Figure S1.*** Trial tree of Experiment 1a. Numbers inside the squares represent the number of trials in each condition. At the top of the tree are represented the total number of trials in the experiment. Trials in each session are divided equally for each session (majority-bivalent, majority-univalent). The proportion of bivalent/univalent trials varies across session and so do the number of N-1 Univalent and N-1 Bivalent trials. Furthermore, trials in each of these cells were equally divided for each level of congruency and task sequence. Branches in red represent conditions that were excluded from analysis. For example, although incongruent trials were also equally distributed between repetition and switch trials, this is not shown in the figure as incongruent trials were excluded from analysis a-priori. In this way, the bottom layer represents the number of trials included in each cell of our ANOVA design.


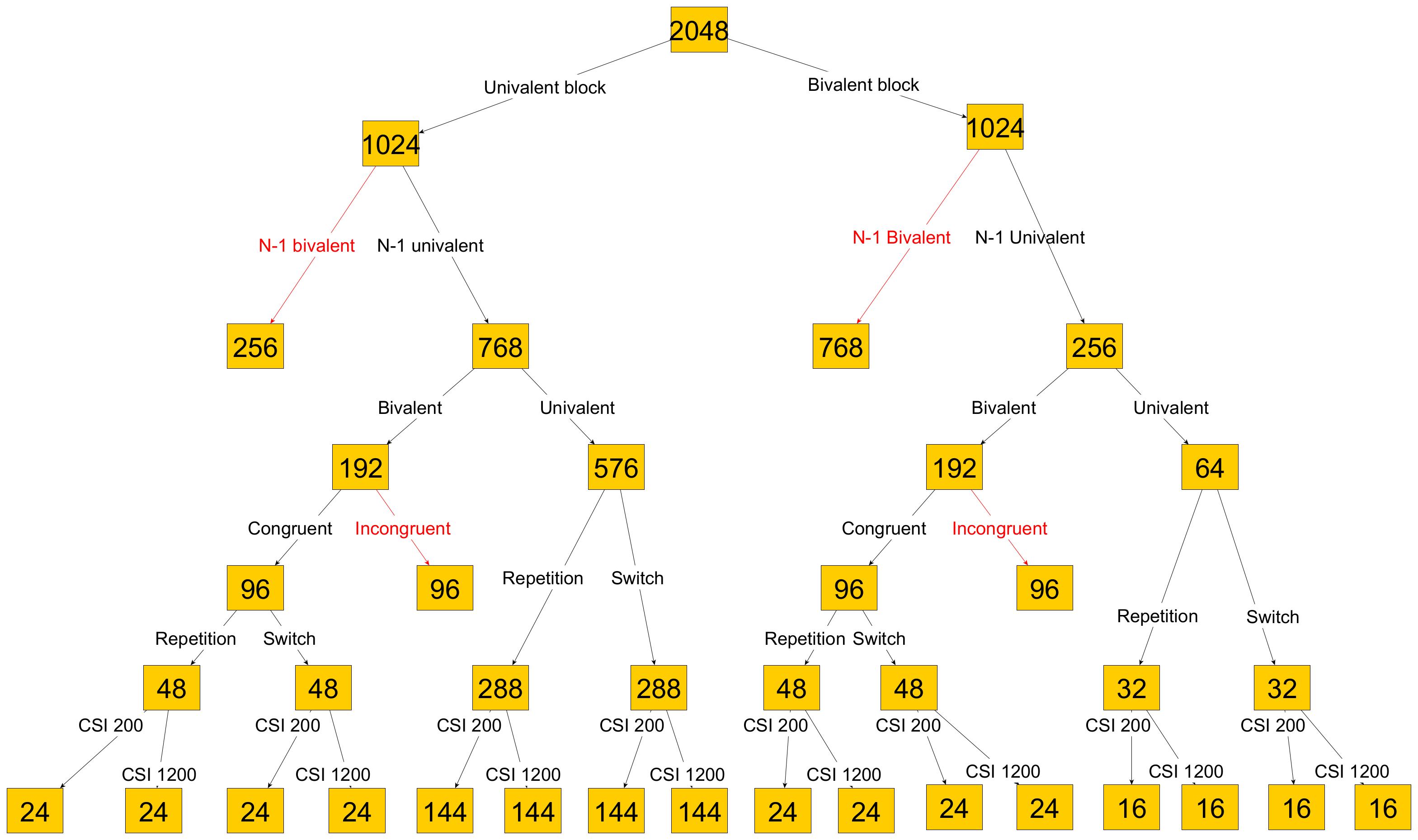


***Figure S2.*** Trial tree of Experiment 1b. Numbers inside the squares represent the number of trials in each condition. At the top of the tree are represented the total number of trials in the experiment. Trials in each session are divided equally for each session (majority-bivalent, majority-univalent). The proportion of bivalent/univalent trials varies across session and so do the number of N-1 Univalent and N-1 Bivalent trials. Furthermore, trials in each of these cells were equally divided for each level of congruency, task sequence and CSI. Branches in red represent conditions that were excluded from analysis. For example, although incongruent trials were also equally distributed between repetition and switch trials, this is not shown in the figure as incongruent trials were excluded from analysis a-priori. In this way, the bottom layer represents the number of trials included in each cell of our ANOVA design.


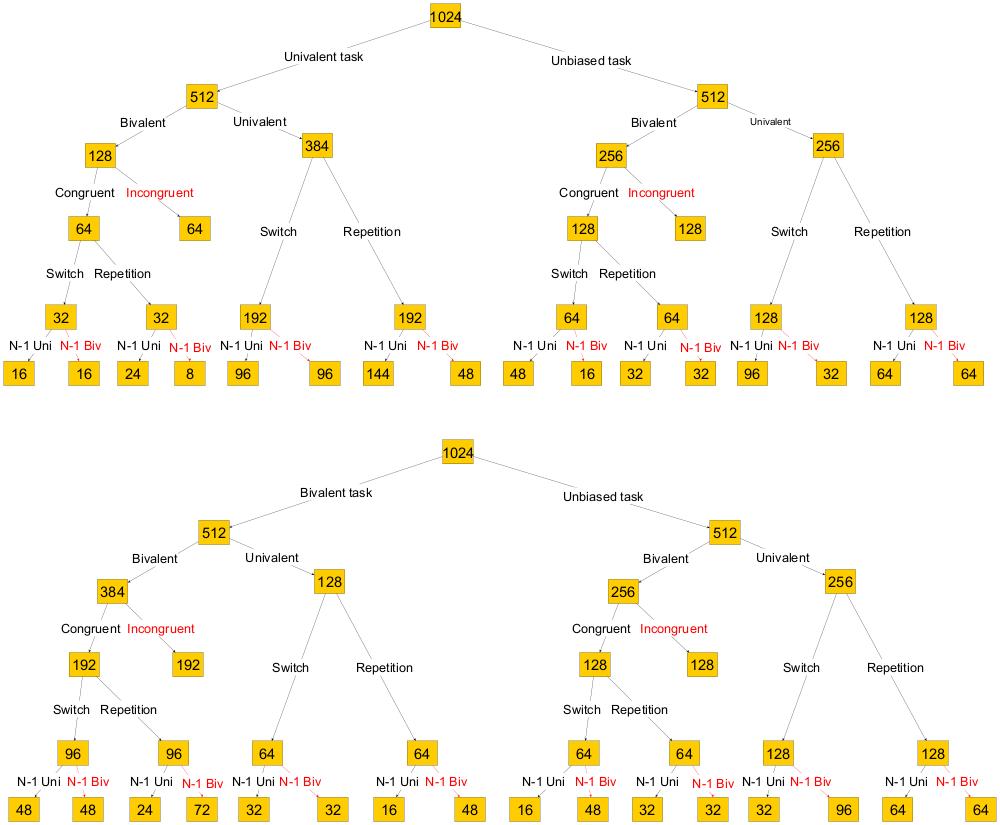


***Figure S3.*** Trial tree of Experiment 2a-2b. Numbers inside the squares represent the number of trials in each condition. At the top of the tree are represented the number of trials in each session. On the top, the majority-univalent session is represented. On the bottom, the majority-bivalent session is represented. Trials in each session are then divided equally for each task (inducer and diagnostic). In the inducer task, the proportion of bivalent/univalent trials varies across session. In the diagnostic task instead, bivalent and univalent trials are equally present in both sessions. Furthermore, trials in each of these cells were equally divided for each level of congruency and task sequence. Branches in red represent conditions that were excluded from analysis. For example, although incongruent trials were also equally distributed between repetition and switch trials, this is not shown in the figure as incongruent trials were excluded from analysis a-priori. In this way, the bottom layer represents the number of trials included in each cell of our ANOVA design.
